# Hybrid IoT Cyber Range

**DOI:** 10.3390/s23063071

**Published:** 2023-03-13

**Authors:** Karl Edvard Balto, Muhammad Mudassar Yamin, Andrii Shalaginov, Basel Katt

**Affiliations:** 1 Department of Information Security and Communication Technology, Norwegian University of Science and Technology, 2815 Gjøvik, Norway; 2 School of Economics, Innovation and Technology, Kristiania University College, Kirkegata 24-26, 0153 Oslo, Norway

**Keywords:** IoT, cyber range, security

## Abstract

The use of IoT devices has increased rapidly in recent times. While the development of new devices is moving quickly, and as prices are being forced down, the costs of developing such devices also needs to be reduced. IoT devices are now trusted with more critical tasks, and it is important that they behave as intended and that the information they process is protected. It is not always the IoT device itself that is the target of a cyber attack, but rather, it can be a tool for another attack. Home consumers, in particular, expect these devices to be easy to use and set up. However, to reduce costs, complexity, and time, security measures are often cut down. To increase awareness and knowledge in IoT security, education, awareness, demonstrations, and training are necessary. Small changes may result in significant security benefits. With increased awareness and knowledge among developers, manufacturers, and users, they can make choices that can improve security. To increase knowledge and awareness in IoT security, a proposed solution is a training ground for IoT security, an IoT cyber range. Cyber ranges have received more attention lately, but not as much in the IoT field, at least not what is publicly available. As the diversity in IoT devices is large with different vendors, architectures, and components and peripherals, it is difficult to find one solution that fits all IoT devices. To some extent, IoT devices can be emulated, but it is not feasible to create emulators for all types of devices. To cover all needs, it is necessary to combine digital emulation with real hardware. A cyber range with this combination is called a hybrid cyber range. This work surveys the requirements for a hybrid IoT cyber range and proposes a design and implementation of a range that fulfills those requirements.

## 1. Introduction

The Internet of Things (IoT) paradigm is a collection of “things” such as devices, sensors, and objects that are connected through a network or the internet to automate tasks and make them easier for humans. These devices can be sensors or controllers for processing temperature, humidity, motion, image, sound, and more, to provide intelligence for a system or a human to perform a task based on the input. These systems are commonly referred to as smart homes, smart health, smart industry/industry 4.0/Industrial IoT (IIoT), smart cities, smart transportation, or smart aviation, in short, smart everything. The devices can include actuators, sensors, power-controlling relays, bulbs, pacemakers, cameras, weather stations, motors, door locks, and so on.

IoT devices can affect the physical world, which is referred to as a Cyber–Physical System (CPS). With CPS, an adversary can cause damage in the physical world through network operations. Not only can it cause physical damage, but it can also collect information about the physical world, including information that affects people’s privacy. As IoT devices take over more human tasks in controlling measurements and actuation tasks, it is essential to ensure high availability and confidence in the installation. IoT devices are often placed in locations without constant power supply and without wiring, which results in IoT devices depending on batteries as a power source and wireless transmission as a communication medium, both with their benefits and challenges.

IoT development is happening rapidly, and it is challenging in the information security field to keep up with the speed. To reduce costs and time during the development and production of devices, information security is not always a priority. Since consumers, especially private ones, are often more focused on price than security, manufacturers have to compete in costs. To increase cyber security awareness among users, developers, and manufacturers, increasing their knowledge within cyber security is a possible measure. To increase these security skills and knowledge in the IoT security field, there is a need for learning and training, and skills should be a desired asset when working within developing, learning, education, forensics, and research. For these purposes, cyber ranges or testbeds are often developed and used.

NIST defines a cyber range as [1]:

*“Cyber ranges are interactive, simulated representations of an organization’s local network, system, tools, and applications that are connected to a simulated Internet level environment. They provide a safe, legal environment to gain hands-on cyber skills and a secure environment for product development and security posture testing."*

*“A cyber range may include actual hardware and software or may be a combination of actual and virtual components. Ranges may be interoperable with other cyber range environments. The Internet level piece of the range environment includes not only simulated traffic but also replicates network services such as webpages, browsers, and email as needed by the customer."*

Malware and cybersecurity attacks have the potential to damage production systems. A cyber range is an environment that ensures isolation from other systems, thereby not affecting production systems. The environment should also be a complete environment where users can be trained, and devices can be tested and demonstrated without being limited by the lack of functionality or realism.

### Problem Description

As previously mentioned, there is a need for personnel training in the IoT security field, and cyber ranges appear to be a solution. While education, reading, lectures, and awareness campaigns do increase knowledge and awareness, hands-on exercises, demonstrations, and training can have a greater impact on learning. Labs and exercises are often used to train all stakeholders in cybersecurity [2]. Cyber ranges can provide both, as defined by NIST above. Cyber ranges must also be designed to provide an environment where the focus is on learning IoT security, rather than spending most of the time administering and setting up the cyber range.

IoT is a challenging field as there are rarely standards available [3]. Even if standards exist, they might not be used or followed at all, or only partially. Cybersecurity exercises are often used to increase knowledge in the field. Learning through practical challenges and discussions complements learning through reading and lectures.

Recently, there have been many proposals for cyber range designs, but there are not many cyber ranges that focus on IoT. While existing ranges can emulate IoT devices, there are challenges that cannot be solved in the virtual realm, and it is not feasible to develop emulation for all devices. IoT testbeds also exist, but their main purpose is not always security, or their design is closed with no open publications. In 2020, Gartner predicted that there would be 21 billion IoT devices in 2025. In 2022, the prediction for 2025 is already 65 billion IoT devices. In 2020, the estimated number of IoT devices was 7 billion, and by the end of 2022, there will likely be more IoT devices than computers on the internet [4].

IoT devices are rarely standardized and require a different approach for almost every device. With a large number of manufacturers and at least the same number of ways to develop firmware and hardware, it is not feasible to have a one-size-fits-all solution. Product life cycles are often short, and the end-of-life for developing and patching for security issues and bugs is earlier than when the customer stops using the devices. Devices are also often forgotten in implementation and are left behind in a network, creating vulnerabilities available to threat actors. Compromised IoT devices can be an issue for the network, the user, other networks, or other people. The Mirai botnet [5] was a botnet of 600,000 vulnerable IoT devices that were controlled for a massive DDOS attack on services on the internet.

A combination of virtual and physical cyber range is referred to as a hybrid cyber range, which combines the best of both worlds. Virtual devices can be emulated or simulated. However, emulating and simulating IoT devices may not always be the best option in a cyber range, as the diversity in devices makes it time-consuming to create an emulation environment adapted to every device type. Therefore, an IoT cyber range should be able to handle physical devices as well as virtual, emulated devices.

Setting up a training environment can be time-consuming when configuring every device for a scenario. When one exercise is complete, and a new team is to perform the same exercise, the administrators of the cyber range are required to set up the same scenario again. Reducing the time spent on preparing a cyber range for an exercise, or managing other phases of an exercise, is time that can be used in developing new scenarios and exercises, as well as reducing costs. Saving costs and man-hours will, in turn, result in training more people in IoT security topics.

According to Vykopal et al. [6], a cyber exercise is costly, especially in terms of time. On the assumption that Cyber exercises increase security awareness and cyber ranges are useful tools for a Cyber exercise, an IoT cyber range could be used to increase IoT security awareness and knowledge. However, a cyber range should be as little resource-demanding as possible while still providing the necessary infrastructure to create realistic exercises and test environments. Keeping that in mind, we investigated the following research questions.

1. Question 1: *What are the requirements for a hybrid IoT cyber range for smart home devices?*
2. Question 2: *How to design and implement a hybrid IoT cyber range fulfilling the requirements stated in question 1?*

In this work, we designed a cyber range for IoT, with a smart home focus, where the cyber range combines emulation and actual physical IoT devices. The cyber range also has a significant automation focus to reduce human interaction when creating exercises and training scenarios, thereby reducing the time spent managing. In the next sections, we share the background and related work relevant to this work. Continuing that, we discuss the methodology used in this work. After that, we present the results from the study, followed by the evaluation and discussion, and conclude the article.

## 2. Background and Related Work

Touqeer et al. [7] conducted a study on the security challenges in IoT smart homes. They categorized the challenges into four layers of IoT: the application layer, perception layer, network layer, and physical layer, each with its unique set of challenges. The application layer contains the applications and services for IoT, while the sensors and inputs from the environment belong to the perception layer. Network software and devices are part of the network layer, and the physical layer comprises the “smart” devices. The researchers also proposed some solutions to address the challenges in each layer.

A more recent paper [8] highlights the need for consumers to increase their awareness and take an active role in their smart home security and privacy. The researchers refer to data from a report by the United Kingdom government that shows consumers lack awareness of what to look for when buying secure products, and there is no marking of products that are considered secure or what their security level is. They propose a platform for IoT security awareness and system hardening advisory that uses concepts such as crowdsourcing and gamification, a solution that is open and available for end-users, retailers, and manufacturers.

Furthermore, Koohang et al. [9] emphasize the importance of end-user IoT security awareness, particularly with respect to privacy, security, and trust. Privacy and security are prerequisites for trust, and IoT awareness is defined as the degree to which users are aware of the basics of growing security and privacy threats of IoT that they may encounter on a routine basis. The researchers found that IoT awareness increases knowledge of IoT security and privacy, which in turn increases trust in IoT. Trust also increases the intention of IoT usage, and awareness and training programs are suggested as activities to increase IoT awareness.

One method of training with defined scenarios, resources, and participants is through cyber exercises. Creating an exercise is time and resource-consuming. Automating cyber ranges reduces the time needed to reset and restart an exercise or scenario. Once a setup is created, a cyber range should be able to be set to its initial state with minimal effort. Vykopal et al. [6] present a cyber exercise life cycle and divide an exercise into five parts: preparation, dry run, execution, and evaluation. In the preparation phase, learning and training objectives are defined, scenarios and background stories are developed, a scoring system is defined, and the technical infrastructure is developed and deployed. A minor test, called Hackathon, is carried out to test the infrastructure. During the dry run phase, the scenario is completed with testing teams to adjust the products from the preparation phase. The execution phase is the planned exercise as it is after the adjustments from the dry run phase. The evaluation phase gathers all information from the previous phases, which is used to improve the exercises, provide learning to the participants, show examples of best practices, and discuss other suggestions for solutions. The execution phase can be repeated for several runs, for several teams, or several retries, requiring resetting the state of the exercise to the same state as before the exercise.

While the exercise phase can take only a few days, the preparation phase can last for several months. Additionally, the dry run phase is likely to be longer than the exercise itself. During the preparation and dry run, the infrastructure is tuned and adjusted. Resetting an infrastructure, especially actual hardware, is a time-consuming task. Automating this task could be beneficial in reducing the overall time for an exercise or for infrastructure testing, allowing more time to increase training, skills, knowledge, and awareness instead of spending time on manual repetition and possibly tasks.

Cyber ranges have many use cases. Päijänen et al. [10] used data from a survey in the CyberSec4Europe project in 2020. Cyber ranges are mainly used for security education (82%), security research and development (72%), competence building (62%), and development of cyber capabilities (51%). A single cyber range could have one or several use cases. The survey listed 11 use cases, and only the ones that covered more than 50% of the cyber ranges are mentioned here. The same paper also shows that 77% of all cyber ranges in the survey support two or more target groups, and 20% of the ranges support four or more target groups. The largest target groups are companies and enterprises (77%), degree program students (23%), and government organizations (23%).

A similar, broader definition of a cyber range than NIST’s definition is provided by Schwab [11]: “Cyber ranges, defined as purpose-built testbeds and experimental research infrastructure, are intended to conduct testing and evaluation, training, and exercises”. A range is a training area, similar to a shooting range for shooting training. It is an area for training in a controlled environment. A cyber range is a training area where cyber is in focus, with training that addresses the challenges that can emerge in the cyber domain. While a range is designed for training, a test bed is designed for testing equipment while developing or in manufacturing. A cyber range can also be used as a test bed, but a test bed needs more functionality to cover the requirements to be as complex as a cyber range.

Cyber ranges have been created since 2008 [12], when DARPA started developing the US National Cyber Range (NCR). The US NCR initiative was started to cover a need for training in cyber network operations. NCR is also used as an abbreviation of the Norwegian cyber range. In 2013, Davis and Magrath from the Australian Department of Defence conducted a survey on cyber ranges and test beds [13]. They identified three main roles for a cyber range: testing of new devices, training to increase the skills and knowledge of persons participating in operations, and research and development. At that point, the training role was the most popular for cyber ranges. At the time of the survey, they noticed a trend where the ranges were moving from simulation to emulation.

Yamin et al. [14] conducted an IoT Smart Home case study in 2018 with a physical IoT device exercise. This exercise was not carried out in a cyber range, but used as a test bed. The paper showed that the exercise significantly improved the skills of the participants. The study also suggested that automation would improve repeatability and reduce resources spent on the exercise. Pure software platforms do not mimic real-world behavior, and therefore realistic exercises should be preferred. The researchers were also suggested to use prefabricated IoT devices, which would reduce the time to assemble an IoT smart home. Another, more recent, paper from Yamin et al. [15] shows that automation could reduce, or even remove, the need for human interaction in White, green, and partially red teams, under the assumption that the system parts are controllable with automation.

**Figure 1:**
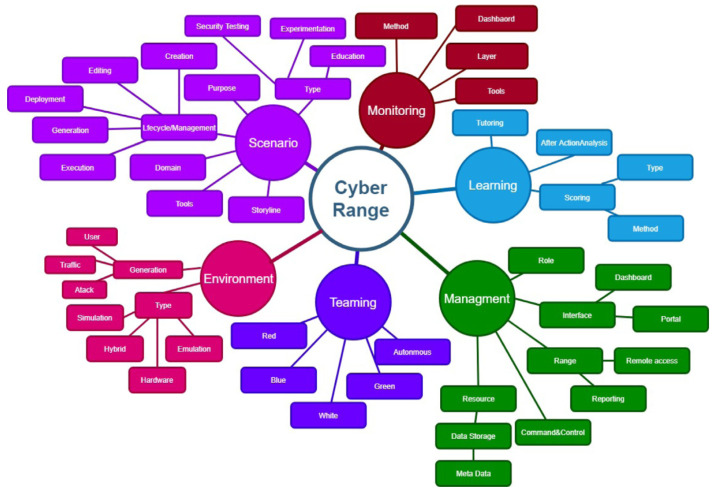
Cyber range taxonomy by Yamin et al. [2].

Yamin et al. [2] conducted a survey on cyber ranges and security test beds, looking into over 100 publications from 2002 to 2018. The paper also discussed requirements for a cyber range. The paper developed a taxonomy for cyber ranges shown in Figure 1.

While developing the cyber range KYPO [16], Vykopal et al. also created a list of requirements. However, KYPO was not initially designed to handle IoT devices, especially not with a hybrid approach.

Industrial IoT, IIoT, is in focus in the paper discussing the design of a cyber–physical cyber range by Kavallieratos et al. [17]. They suggest a reference architecture for Cyber–Physical-Systems (CPS) with a hybrid approach and also perform an assessment of existing testbed features to a list of expected features in a testbed developed for security research. A more recent survey in cyber ranges and testbeds is conducted by Chouliaras et al. [18]. The authors survey 10 developments and also conducted interviews with the system owners to provide insights into modern cyber ranges. The paper gives a good insight into the state-of-the-art within the field.

Al-Hawawreh et al. [19] developed a design and implementation of an IIoT testbed for security researchers. The testbed, called Brown-IIoTbed, is a hybrid testbed with virtual servers and physical installations. The IoT devices used in the testbed are used as industrial devices, although not all devices are industrial grade. The approach has relevance for this work as it contains a physical dimension and has several IoT communication channels. However, this testbed design does not have a provisioning focus, nor is it designed as a cyber range.

The “Poor Man’s IoT Testbed” by Muños et al. [20] shows a flexible and scalable approach to creating a testbed using reasonably priced off-the-shelf products for IoT devices and communication protocols. This physical installation consists of a Raspberry Pi controller communicating with four remote microcontrollers and their sensors, which are nicely installed in a glass dome. While the design could be adapted, at least partially, to a cyber range, it currently lacks provisioning and automation, and its implementation is physical-only.

The IoT-CR cyber range [21] is a hybrid cyber range consisting of 20 Zolertia devices called RE-MOTE, virtual devices, a resource engine, and a front-end engine. The physical devices are controlled by the server via USB interfaces, and the virtual devices use Contiki-NG embedded RTOS executed on top of Cooja. The paper describes a demonstration of an autonomous scenario called “Pass the Token”.

CyRange [22] is an educational IoT cyber range for forensics that comprises four blocks: an IoT system simulation, a middleware/database, a learning management system, and a forensics workstation. The forensics workstation is set up with a forensics toolset and visual decision support tools. This cyber range is developed for live digital forensics and training people for those tasks. In IoT forensics, which is based on digital forensics, there are three zones: the internal network, middle network, and outside/external network. IoT includes several digital forensics sub-types, such as cloud forensics, network forensics, and device forensics.

A Raspberry Pi cyber range was created to teach web attacks in [23]. Raspberry Pi is a mini single-board computer often used in IoT projects because of its easy-to-use General-Purpose Input/Output (GPIO) interfaces. While the cyber range is not IoT-targeted, it uses IoT devices to create a cyber range and is able to scale the solution by adding and removing Raspberry Pis from the cluster. The project uses a low-cost cyber range to do security training. CyberIoT [24] is a conceptual design of a cyber range for IoT. However, the design is unclear on how the IoT devices interact with the cyber range, and it is assumed that the IoT devices are emulated. Emulating is not always the best solution for IoT training.

Cyber ranges have an element of a testbed as defined by Schwab and Kline [11]. Several testbeds are developed for many purposes in IoT, such as a network testbed, Testbed@TWISC [25], a testbed for eHealth [26], a SCADA testbed [27], an IoT automated testbed [28], and many more. Testbeds, however, lack the complete network and administrative infrastructure that cyber ranges have to be used as infrastructures for cyber exercises. A testbed framework for wearable IoT devices [29] has a design requirement for data forensics analysis, where data extraction should be performed by side channels such as USB and JTAG, using ADB, for example. Another requirement is to have an array of simulations to manipulate the IoT sensors, e.g., GPS simulator.

## 3. Research Method

**Figure 2:**
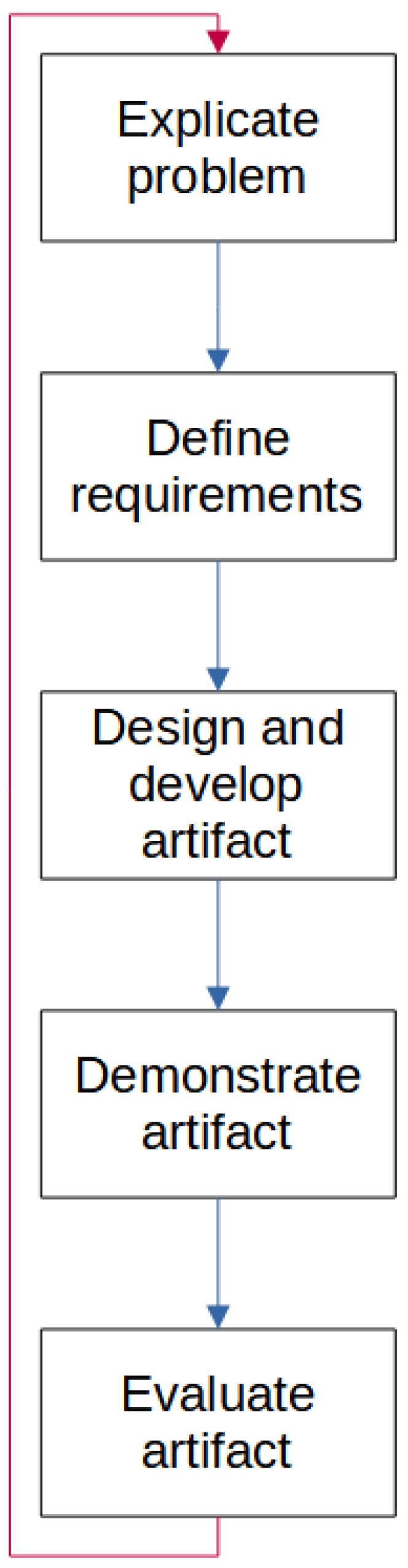
Activities in design science research.

Empirical research aims to describe and explain, while design research also seeks to change something in the world. Design research develops artifacts to improve something. Design science is a design research framework that has been widely developed and used in the information technology domain. It is a methodology that can incorporate several other methods in each of the activities in the framework. The methodology used for this work is Design Science Research [30]. Design science aims not only to design something but also to generate new knowledge, utilize existing and well-accepted scientific knowledge, and make the knowledge available to other researchers. In design science research, an artifact must be defined and handled through some defined activities. IT artifacts are broadly defined as constructs, models, methods, or instantiations. Artifacts can fall into one or more categories. The five main activities in Design Science Research, as shown in Figure 2, are:

- Explicate the problem: investigate and analyze a practical problem and its importance.
- Define requirements: outline a solution to the problem and elicit requirements.
- Design and develop an artifact: fulfill the requirements.
- Demonstrate the artifact: prove feasibility in one case.
- Evaluate the artifact: assess how well the artifact solves the explicated problem and fulfills the defined requirements.

Each activity has an input from the previous activity, an output to the next activity, and will also consume controls and resources. Controls are in the form of strategies and methods, and resources are in the form of knowledge and workload. In the explicate the problem activity, there are three sub-activities:

- Define precisely.
- Position and justify.
- Find root causes.

To explicate the problem, a problem description needs to be precise and justified. The problem should be of general interest, while investigating the underlying causes for the problem. For this purpose, a literature search for existing IoT cyber ranges and security test beds was necessary. The project starts with a literature search of the state-of-the-art published work in IoT cyber range or IoT Security testbed development and designs. Papers were searched for in NTNU Oria, Google Scholar, and IEEE Explore. Search strings included IoT, IIoT, industry 4.0, smart home, cyber range, test bed, or testbed. It is also relevant to study the “future work” section in all papers to find what is needed in an IoT cyber range, and whether there are solutions for automating, and if not, is there room for improving a cyber range setup with more automation. Some cyber range surveys, without IoT focus, were studied to gain more foundation on the trends in the cyber range, regardless of what they are designed for. Existing cyber range surveys also show what cyber ranges are used for and the benefits of cyber ranges. The surveys of cyber ranges and test beds also guided towards the existing cyber ranges and test beds in the IoT, especially smart home, domain.

Since published papers are often published some time after they are written, it can be challenging to have an overview of the state-of-the-art projects and the challenges/needs of those projects, or what is missing in them. Since this project aims to develop a cyber range design for IoT, an approach to obtain the most current best practices and practical challenges was desired. To gain a better understanding of the challenges within the domain, a questionnaire was developed for surveying the cyber range, especially IoT cyber range, community. The recipients were selected from the cyber range research and user community in Europe, and were also requested to forward the questionnaire to others who may have contributions to the project. The recipients were selected from the authors in the literature review and also suggested contacts within the supervisors’ network.

In design science, the “define the requirements” activity has two sub-activities: outlining the artifact and eliciting the requirements. The artifact in this project is a model of an IoT cyber range, as well as a partial instance of the same cyber range. As the time for this project is limited, finding automation solutions is prioritized.

To define the requirements for a hybrid IoT cyber range, the results from a literature search and the earlier-mentioned questionnaire were used to create the requirements for this project. The requirements are discussed in Section 4.1. The same papers as in the previous activity were used as the basis to define the requirements for an IoT cyber range. The results from the questionnaire survey were also expected to contribute to a larger extent than they actually did. The requirements are summarized in a qualitative analysis of the information from the literature and the questionnaire.

The “design and develop” activity in design science has four sub-activities: imagining and brainstorming, assessing and selecting, sketching and building, and justifying and reflecting. To design and develop a hybrid IoT cyber range, surveys in the cyber range domains were conducted to show the solutions chosen by others when designing a cyber range. This helps in getting a feel for what technology is available, how others assessed the technology, and choosing the best solution for this project. As much of the technology is new for this project’s author, a large amount of time is spent learning the pros and cons of the technical and practical solutions that emerge from the surveys. Learning the capabilities of the technology should give ideas on how the different solutions can be used to solve an IoT cyber range. After having a good grasp of the capabilities of the technologies studied, both those used by others and those found by the author, a design should emerge by choosing those functions that seem to fit the best. All sub-activities in design and develop are discussed in the design and implementation section. All sub-activities overlap to some extent in those sections.

“Demonstrating” as a main activity, has two sub-activities: choosing and designing a case and applying the artifact. Demonstrating the cyber range shows a proof-of-concept from the developed solution and shows that the problem described has a feasible solution. This activity includes implementing known attacks for IoT devices and showing that the artifact can be useful. Although Johannesson and Perjons [30] state that the artifact should be demonstrated for one case, this project tests the cyber range for two test cases.

“Evaluation” has three sub-activities: analyzing context, selecting a goal and strategy, and carrying out the evaluation. The main goal of evaluation is to determine whether the artifact solved the defined problem and how well it solves the problem.

The evaluation must demonstrate how well the end product covers the requirements set earlier and how effectively the problems described have been solved. An evaluated artifact of a **DSR** process could be improved as a new problem description, thus the methodology can be an iterative process, a cycle, as shown in the red line in Figure 2. For this project, the DSR activities are only performed in one sequence. The cyber range is evaluated against the requirements found in the survey, as well as showing the gains in resource usage while preparing the scenarios for dry runs, execution, and re-runs. The artifact are evaluated in the discussion section, where the evaluation context, goals, and strategy are described before the evaluation is conducted.

## 4. Requirements, Design and Development

This section discusses the requirements for a hybrid IoT cyber range in the requirements section, suggests a design in the design section, describes a possible implementation as well as demonstrates use cases in the implementation section. The requirements section first shows the requirements found from the literature search, then the results from the survey, before synthesizing the requirements. IoT awareness is increased by knowledge [9]. Knowledge is increased in cyber exercises [31]. Further, several papers support that a cyber range provide beneficiary tools for carrying out cyber exercises.

### 4.1. Requirements

According to Yamin et al. [2] a cyber range must cover 8 functional architecture components:

- Portal.
- Management.
- Training and education module.
- Testing module.
- Scenario.
- Monitoring.
- Run time environment.
- Data storage.

Yamin et al. also surveyed the future needs to a more efficient exercise lifecycle management and suggested more automation as solution. For scale-ability SDN and use of containers are suggested technologies. Federation is also pointed out as a future direction, as well as user interaction simulation to reduce human interaction and thereby increasing efficiency.

Kavallieratos et al. [17] developed a reference architecture with a control center module, physical components module, virtual components module, cybersecurity defensive mechanisms module. While Yamin et al. has a more general description Kavallieratos et al. has a more hands on approach mostly in the run time environment component of Yamin’s description.

The requirements in the Kavallieratos model are flexibility, scale-ability, isolation, interoperability, cost-effectiveness, built-in monitoring, easy access, adaptability and shareability. While Yamin does not explicitly state requirements for cyber ranges, some components in the architecture cover the same requirements.

When designing KYPO [16], the authors had some important requirements: flexibility, scale-ability, isolation vs. interoperability, cost-effectiveness, built-in monitoring, easy access, service-based access and open source, which to some degree are the same requirements as Kavallieratos has described.

The tier approach from AL-Hawawreh and Sitnikova [19] with an edge tier, platform tier, and enterprise tier can give a new dimension for a IoT cyber range design. They suggest nine features for comparison with other test beds in their implementation of the Security Testbed for **IIOT**:

- Usability.
- Fidelity.
- Heterogeneity.
- Flexibility and scalability.
- Federation.
- Safety, reliability, and resilience.
- User interfacing.
- End-to-end testbed.

Ukwandu et al. [32] surveyed 44 cyber ranges as well as many cyber attacks used in scenarios. They also show some future trends, technologies, and uses of cyber ranges, which are relevant to look into while designing a cyber range. The trends they list are: real-time auto-configurable systems, smart, mobile, and integrated technologies and training with augmented reality. The technology trends they list are 5G/6G technologies and more containerization. The application area trends in the paper are pointing towards smart **CPS**, smart cities, and industry 4.0, and lastly, aerospace and satellite industries. Ukwandu et al. also suggested a taxonomy for cyber ranges not very different from the taxonomy from Yamin et al.

Siboni et al. [29] built an IoT testbed for wearable IoT devices. During design, they listed several requirements for an IoT testbed:

- Should be able to handle a wide variety of devices from different categories.
- Should also be able to emulate different testing environments.
- Should support Security tests, and the paper suggested 13 different security tests to support.
- Should simulate actuation’s and signaling.
- Should support of the most common wireless and wire communication channels.
- Should be able to process and analyze relevant communication protocols.
- Should be able to extract data from the tested devices, preferably on side channels.
- Should support management and report mechanisms to control and and manage test flows.
- Should support user intervention and automation capabilities.
- Should be plug-able.

### 4.2. Results from Questionnaire

The questionnaire, mentioned before, that was distributed to a selected recipients, resulted in a low response count. The 22 recipients are from different universities, research organizations, and cyber ranges in Europe, and in total, 4 people responded answering the questions. One other recipient responded that he/she could not make time to answer the survey. The questionnaire was designed as a multiple choice questions and with the alternatives were chosen after studying literature on cyber ranges and testbeds, both IoT and others. Some questions also were open ended questions. This section describes the questions in the survey as well as the results from the questions. The questions were designed so the respondents would not have to reveal any secrets about their own implementation.

First question asked what type of cyber range the respondent has: virtual, physical or hybrid. Three of the respondents have a hybrid (virtual in combination with physical) cyber range, one has virtual only. The second question was about what category of IoT devices the cyber range supported. Two of them support smart homes, two support smart health, two support smart transportation, two support smart grids, and two support IIoT/smart industry. Only one supported smart cities. Third question was the usage of the cyber range: The use of the cyber ranges are **IRT** training, penetration testing, and forensics training. The open answer questions about what the functions and interfaces were unanswered.

The next questions were about the technology used in the cyber ranges. The technology used for orchestration is mostly self-developed, one uses OpenStack Heat. For management of the cyber range, three of them use self-developed tools. One uses OpenStack and another uses vCenter in combination with self-developed tools. One flaw in the suggestions in the published questionnaire was that Ansible was not listed as an option, though it probably would not make much of a difference. Defining scenarios are for two of the respondents made in the JSON format, and another uses self-developed tools for this. Emulations are conducted in Virtual Box, OpenStack, VMWare, and in self developed tools. Two respondents use VirtualBox, while one use VMWare in combination with self-developed tool. Two of the respondents use tools for simulation, one has a self-developed tool and the other uses OPNET. For monitoring one uses ELK in combination with WAZUH. WAZUH was a new suggestion to the original listing in the questionnaire. Another combines Wireshark with tcpdump, the third combines ELK, Bro/Zeek, Snort, Wireshark, and tcpdump. In retrospect, Tshark should be included as an option but can fall into the Wireshark selection. In addition, for data representation JSON is the preferred tool for three of the respondents, the fourth uses a self-developed solution. As the project is for IoT, the questions should have been more precise that the data representation is for the IoT and physical world representation. Creating data is, as the previous point about data representation, meant for the simulation of real world and IoT data. The question might not make this clear enough. Three of the respondent’s answer self-developed tools for this and the fourth answer “Klai linux” in the “other” field. This is most likely to be a typing error and meaning Kali Linux.

The comments from the respondents in the question of what the top three functional requirements are for IoT cyber ranges that differ from conventional/general cyber range designs are:

- Mapping digital twin I/O on a low level.
- Cross-platform deterministic human interaction.
- Integration of advanced techniques such that it achieves steganography detection and exercise.

The main challenges of cyber range and security testbed implementations, especially IoT focus, are

- Diversity of the IoT components.
- Low-level performance in SW implementation vs. high-level portability.

When asking for the challenges generating sample data, both benign and malicious there was only one answer: “Cross-platform adaptation” Only one answer came for the question about own IoT cyber range drawback is “**AI** utilization”.

The one suggestion on how to solve challenges and functional needs was: “adopt the changes in functional requirements whenever possible”.

The questionnaire form, as sent, is available in the appendix.

#### 4.2.1. Requirements Summary

Analyzing the information of requirements from the literature review and the results from the questionnaire result in these design requirement categorized for this cyber range:

- Flexibility, scale-ability, adaptability, and interoperability.
- Shareability and open source.
- Fidelity.
- Isolation, safety, resilience, and reliability.
- Cost-effectiveness.
- Built-in monitoring.
- Easy access, usability, and user interfacing.
- Service-based access.
- Heterogenity, handle diversity in IoT, and emulating digital twin.
- End-to-end testbed.

Flexibility and scale-ability is important to create scenarios as close as possible to real life implementations. The cyber range should give training that resembles real life environments whether training for large or small environments. Adaptability and interoperability are requirements to the system to be able to change with minimal efforts, either to integrate with other systems or to change the internal functions in managing the cyber range or within the scenarios.

As Vykopal et al. state for the KYPO implementation, the platform should reuse suitable open-source projects and release its artefacts under open source license. Use of existing open source projects gives others opportunities to use and reuse this project’s artifacts. This is important to make the training for security as manageable as possible for as many as possible, in common available and affordable tools. Others refer to this requirement as shareability.

Fidelity requirement refers to follow standards. Until recently there were not developed standards for cyber ranges. With the work of Yamin et al. and later Ukwandu et al. taxonomies were created. Yamin et al. also developed a list of functional requirements for a cyber range. Some standards exist for IoT communication protocols, but the payload is often up to the developer. Zigbee Home Automation Profile is one example of existing standard for communication at an application level. Seljeseth et al. [3] also suggested data formatting framework for IoT devices, UIoT:FMT, the paper also addresses some of the challenges of standardization within IoT.

Isolation, safety, reliabilty, and resilience are requirements so that the cyber range itself is not vulnerable to anything happening in the scenario. In addition, vulnerabilities and malware in the scenarios should not gain access to other networks than intended, e.g., Internet. The cyber range infrastructure should not lose any management functions due to resource depleting in the training area, e.g., **DDOS** attacks or process-intensive malware and such. When dealing with wireless signals, [29] also isolated the wireless signaling from the surroundings with a special built, shielded room.

IoT is emerging quickly; one of the reasons is that IoT devices are affordable to even normal households. A cyber range should also be cost-effective to give as many as possible the possibility to use the benefits of a cyber range. Using openly available software is one measure to achieve this requirement, emulation is another possibility (with the before mentioned challenges), and using devices that require little modification could reduce the time to set the environment up. Automating tasks that can be automated will reduce the time to obtain the range ready for exercise, and even reduce the total labor time in setting it up. Automating and automatically controlling the physical devices within the exercise zone is a large benefit in reducing labor, and thereby increasing cost-effectiveness.

The service-based-access requirement is to some extend reasoned for reducing costs and some for providing a cyber range as a service to reduce the needs for implementing many ranges. The interoperability requirement is about consuming services from providers, while this requirement is about providing services.

Since the IoT device implementation diversity is large [33], the protocols are many and the lack of standards or the lack of will to follow standards the cyber range should require heterogeneity. The system should be able to emulate a large collection of devices as well as be able to support many types of protocols.

Al-Hawawreh and Sitnikova developed an end-to-end test bed and used this feature as comparison. A cyber range should be able to provide the end-to-end communication for IoT devices. Many IoT devices communicate directly to a service provider in the cloud and is managed through external providers. Implementing services, such as a Home Assistant (https://www.home-assistant.io/ (accessed on 16 February 2023)), simulate, to some extent, the service provider of IoT management from a user perspective where the user can operate his or her smart home through a web interface or through an mobile device app.

### 4.3. Technology

A design depends on the technology available. One of the requirements is to use open source software and to be cost effective. This section discusses the technologies that make the design feasible and some of the challenges that the design has to overcome.

#### 4.3.1. Emulation

Having every possible IoT device available for training or testing is not feasible. The diversity in devices is too large. Running IoT firmware outside its hardware environment requires emulation. Emulating an IoT device seems to be a possible solution to reduce storage and costs. Once developed a virtual infrastructure with digital models [34] of the IoT devices, the devices can be shared with others.

The benefits of emulation are many. Enquiring a large stock of devices require storage space, is expensive and a lot of effort, especially when devices are end-of-production. Running device emulation, with several devices on a commodity computer saves all that and computing power. The benefits are also environmental as the devices at some point has to be disposed.

It is a challenge to emulate a large specter of IoT devices. While the IoT devices are all microcontrollers and computers, they have a large diversity in architecture and compositions. Even within the ARM architecture there are 19 product families, which ARM themselves produce, although lately only Coretex families are developed. Qualcomm also develop CPUs using the ARM instruction set, and several other vendors. To make things even more complicated, the CPUs are often embedded into **socs** (System on Chip). SoCs or CPUs are often part of a system with integrated functions on one board or even with peripherals. Some multipurpose device examples are Arduino boards or Raspberry Pi boards. In addition, when emulating IoT devices, one must be aware that physical IoT devices have more constraints than emulated devices, e.g., limited processing power, limited power supply, etc.

To emulate a complete system, it is not sufficient to emulate a CPU, all connected peripherals must be emulated. According to Zaddach et al. [35] there are three possible emulation solutions: complete hardware emulation, hardware over-approximation and firmware adaptation. Zaddach et al. proposed a system called Avatar^2^ where the CPU is emulated with QEMU and **JTAG** is used to communicate with the actual hardware.

While there are many emulators for each specific architecture, e.g., for AVR: Avora, AVRS, simavr, SimulAVR, atemu, GNU AVR simulator, IMAVR and probably many more, some emulators support more architectures. Most solutions in the literature use QEMU as emulation platform, QEMU has support for ARM, AVR, x86, RISC, SPARC, and more architectures and already has support for many SoCs and boards. QEMU is also a hypervisor supported by OpenStack.

#### 4.3.2. Simulation

As IoT devices main task is to represent something in the real world, a digital model of the IoT device is not connected to a real world sensor. This information need either to be simulated with random data, information from a data model or remotely connected to a real world sensor.

#### 4.3.3. IoT Communication Interfaces

IoT devices can be sensors and actuators installed in locations where it is not desirable to install cables, either because it would be not feasible to install or it is easier or more cost-efficient not to install. Thus, IoT devices often have to rely on wireless communication to own control systems. And since the devices are not cabled and need power, the devices must rely on batteries. As the power supplies are constrained there is a wish to reduce power consumption and therefore some protocols are developed for constrained devices. There are wireless protocols such as Z-Wave, Zigbee, Bluetooth Low Energy (BLE), LoRaWAN, etc. Some protocols are long range and some for short range. Where power supply is not a problem, IoT devices often use WiFi since the infrastructure already exists and also reduces the complexity for the user. Where network cables exist, IoT devices can also obtain the power supply through PoE; thus, wireless and power challenges are not an issue. Network vulnerabilities still can exist.

The SoCs on the IoT devices often come with some capabilities on the chip. The chip can implement functions as **uart**, **jtag**, **spi**, and **swd**. Universal Asynchronous Receiver–Transmitter (**uart**) is also often referred to as serial port. The UART is a hardware device for sending and receiving serial data over three wires, one for transmitting (TX), one for receiving (RX), and reference level/ground (GND). Often there is also a fourth wire with constant reference voltage (VCC) or as power supply. What the UART is used for depends on the firmware of the device, often the UART communicates what the console would do if there was a terminal present.

JTAG is an industry standard for verifying and testing PCBs after manufacture. JTAG is named after the Joint Test Action Group, which developed the standard. JTAG is a debugging port on a PCB with serial communication to Test Access Port (**tap**) on the each chip. JTAG provides a possibility to debug CPU’s at a machine instruction level, such as stopping execution and reading registers. JTAG also allows reading and writing firmware on the non-volatile memory, such as flash memory. One of the main initial functions of JTAG was boundary scan testing. Boundary scan gives an opportunity to read and set inputs and outputs of each pin on the chip.

The JTAG connector pins are Test Data In (TDI), Test Data Out (TDO), Test Clock (TCK), Test Mode Select (TMS), and Test Reset (TRST). The TAPs are daisy chained with TDO on one TAP connected to TDI on the next TAP. The last TAP TDO is connected to the JTAG connectors TDO. This means that when reading the first TAP, the data must pass through all subsequent TAPs before reaching the TDO connector. The TCK speed decides how fast the information flows through the TAPs. The slowest TAP limits the JTAG TCK speed, but typically, 10–100 MHz is accepted as the clock speed [36]. Holding the TMS pin state unchanged also keeps the test mode unchanged. Cycling the TMS changes the test mode on the TAP. The chip can be unaffected by the TAP or be set to behave in a specific way. A System Reset (SRST) pin is also a useful pin that allows resetting the entire system. An alternative specification of JTAG is a reduced pin count JTAG (IEEE1149.7) called cJTAG for compact JTAG. cJTAG has two pins with Test clock (TCKC) and Test Serial Data (TMSC) where the TAPs are connected in a star topology.

Serial Wire Debug (**swd**) is also a two pin debugging interface developed by ARM [37]. The SWD pins are Serial Wire Clock (SWCLK) and Serial Wire Input/Output (SWDIO). All SWD operation sequences consist of two or three phases: Packet request, acknowledge response, and possibly a data transfer phase. The SWD, similar to JTAG, can give access to registers, memory, and internal buses.

SPI is a synchronous serial communication designed by Motorola in 1979. SPI uses a master–slave architecture and is capable of full duplex. Multiple slave devices are possible through a slave-select (SS) wire, e.g., the Arduino Pro Mini (Atmega 328P) makes it possible to program through In-System-Programming (ISP/ICSP) via SPI connections as it is embedded in AVR SoCs.

#### 4.3.4. IoT Wireless

IoT devices often use wireless communication. There are several standards for wireless communication. As sensors often are placed in locations without access to constant electricity, devices are powered with batteries. Wireless communication in combination with limited power supply do create some challenges. The amount of data sent over the air must be reduced to reduce power consumption, the amount of awake time is often also reduced. Some manufacturers use proprietary protocols for communication; however, as the IoT wireless standards become more mature the developers chose to use the already developed standards to reduce costs and development time. Many microchip vendors also integrate protocols into their products and can offer a single System-on-Chip (SoC). Some wireless protocols used in IoT are standards such as Wi-Fi, Zigbee, Z-wave, Bluetooth, and many proprietary protocols modulated on top of 433 MHz or IEEE 802.15-4.

Zigbee is a well-known wireless specification of protocols built on top of IEEE 802.15-4 specification. IEEE 802.15-4 specifies wireless communication for LR-WPAN (Low rate wireless personal area network) and is also the basis for 6LoWPAN. As the name LR-WPAN indicates, the data amount is small, the network is wireless, and the scope is at personal-area level. Zigbee is built for low power consumption, relatively large networks, and to utilize other nodes in the same network to extend the range of the network. Zigbee security is criticized as the network-registering process in the basic security model requires the network key to be transferred unencrypted over the air. The network key is necessary to join the network and must be shared by all devices. Another layer of security is the link key, which encrypts the traffic before the devices acquires the network key. There is a default global trust center link key defined by the Zigbee Aliance, which normally all new devices know of. The default key is “5A6967426565416C6C69616E63653039”. A third key is the master key, which is a key that encrypts traffic between two nodes, and it is a long time key between the devices that is established in a key exchange phase between the devices. A Zigbee device can have the role of a coordinator, which is in charge of the network, a router, which can be an end device, but is also responsible to forward traffic, or an end device, which has no obligations but to itself. A router cannot sleep as it must route traffic between end devices and the coordinator, or possibly between end devices and routers, or between routers and routers. Routers and coordinators should not be powered by batteries as they cannot sleep to save power.

#### 4.3.5. Linux-Based IoT Boot Process

On embedded platforms, the boot process is bit different from standard x86 computers. When the device is powered up, the **SoC** reads the internal ROM code. The ROM code is hardcoded into the processor. The ROM code instructs the processor to load the next stage, the first-stage bootloader. The first stage boot loader does some basic hardware initialization, e.g., memory, and loads the second stage boot loader. The second stage boot loader sets up media such as NAND, SD card, network, file systems, and sets up the root file system and start to load the kernel. The kernel handles the rest of the peripherals, handles memory and process management, and mounts the root file system. Both the first and the second-stage boot loaders can be overwritten, as well as the kernel and the root file system. The operating system in the Linux-embedded devices is loaded to memory and all changes in the root file system are performed in the memory. The devices rarely have functions to write changes made in the file system back to the flash storage, and after rebooting the changes are lost. This is except for the configuration changes made to the devices, which is written to a different memory region and is also the area that is wiped during a reset to factory default settings.

On Raspberry Pi the ROM code is called stage 1; this stage is executed on the GPU. The GPU loads the stage 2, bootcode.bin, from the SD card into the L2 cache. Stage 2 enables the SDRAM and loads boot stage 3, loader.bin, into RAM and runs it. The loader then loads the start.elf to boot the operating system. Newer versions of Pi skips the 3. stage, and the bootcode.bin loads the start.elf.

### 4.4. Design

This design seeks to fulfill the requirements for a complete cyber range. The architecture components from Yamin et al. (as discussed before: portal, run-time environment, management, training and education, testing, scenario, monitoring, and data storage), are suitable to cover the components used in cyber ranges that are mentioned in the previous sections. As Yamin et al. suggest a unified architecture, and the architecture fits, it is wise to use it for the work. There are some components that can be general for all types of cyber ranges, no matter the purpose. As this work focuses on a hybrid IoT solution there are components that must be specific. The components differing from a general cyber range to an hybrid IoT cyber range are the run-time environment, monitoring, and scenario. This section discusses how this design is solved in the various components.

#### 4.4.1. Portal

**Figure 3:**
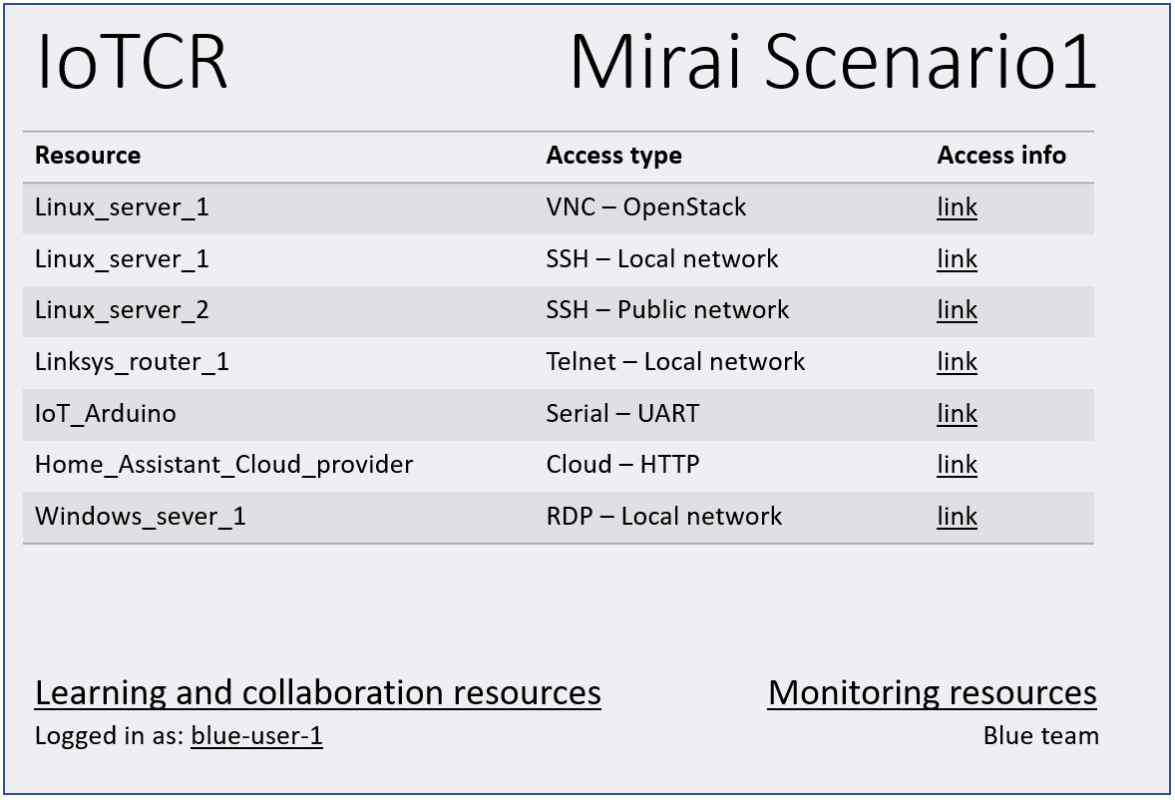
Portal view example.

A portal should give the users in all teams the tools to administer and use the cyber range. This project does not focus on the portal, but acknowledges the need for a portal to access the resources needed to be a part of a team in the cyber range. These resources could be scenario provisioning, credentials, network access for management, computer console access for the users, etc. The portal also gives access further to the network, such as management interfaces of the cloud solution and console access to the management servers, access to learning platforms, etc. The portal should handle authorization and authentication of the users. The requirements in the previous section, i.e., easy access, usability, and the possibility for user interfacing, should be, to a large extent, covered by the portal component. A suggested portal view for one of the exercise participants is shown in Figure 3, e.g., OpenStack provides console access to instances over VNC.

#### 4.4.2. Training and Education

Training and education module should give learning materials to the users; this function should also cover the possibility to give grades and feedback while learning. **Moodle** (https://moodle.org/ (accessed on 16 February 2023)) is a free and open source learning management system with course administration. Moodle is php-based, modular, and has a wide variety of plugins. Moodle could serve as a portal for the cyber range and be an identity provider for the other services within the cyber range as well.

#### 4.4.3. Run Time Environment

This component is the main focus of this project. The design should able to both emulate and run actual hardware. The goal is to automate the provisioning and orchestration as much as possible. The provisioning is to be made in the virtual and physical platform. **Ansible** (https://www.ansible.com/ (accessed on 16 February 2023)) is an open source software for automation and provisioning, configuring, and installation tool. Ansible supports Linux, Windows, and Mac. It is an agent-less software, using remote connections to the target for running commands. Ansible is used as the base software for the provisioning and Ansible supports and can control many other solutions. Ansible controls the orchestration of the cloud and on-site system. For provisioning the system Ansible controls Terraform which in turn manages the OpenStack functions.

**Terraform** (https://www.terraform.io/ (accessed on 16 February 2023)) is an open source orchestration, infrastructure-as-a-code software developed by Hashicorp. Terraform support several cloud platforms such as AWS, Google Cloud, Azure, and OpenStack. Terraform provisioning is performed by declaring the infrastructure and its providers through HashiCorp Declaration Language. The definitions are generated by Ansible with templates bases created by the cyber range administrators. Terraform works similarly to OpenStack Heat, but Terraform has plugins for many more systems.

**OpenStack** (https://www.openstack.org/ (accessed on 16 February 2023)) is an open source cloud computing platform for managing private and public clouds. OpenStack has modular design providing many components. OpenStack Ironic is a component for bare metal provisioning of physical hardware as opposed to virtual machines. For this project, an OpenStack cloud is used; however, since we also need a hardware platform, then OpenStack Ironic is used. The cloud solution used in this project is managed by an external party, so integration with Ironic is not an option; to be able to have flexibility to use the solution with other cloud providers, we chose to install a separate Ironic platform and orchestrate this through Ansible and Terraform. A cloud installation on an existing cloud is referred to as a cloud-in-cloud solution.

Linking the virtual world with the physical installation should be fast, reliable, and secure. Wireguard (https://www.wireguard.com/ (accessed on 16 February 2023)) is a free open-source VPN software, which shows good results [38]. Wireguard is designed to be easy to use, have good performance, reduce overhead, and reduce attack surfaces. Wireguard communicates over UDP.

#### 4.4.4. Networking

A cyber range must have one or more networks to exercise in. As stated in [2], teaming is an important part of a cyber range, and the teams in training must have their own network(s) to defend or to attack from. Depending on the needs for the scenario and the what and how the teams organize, some possible network needs are: Internet, red team network, blue team network, provisioning, and management networks. The provisioning network is for provisioning the devices over network, whereas the management network is for administering the devices as well as giving the automation processes access to the infrastructure nodes. Networks need to be separated to cover the isolation requirement and to create realism; to ensure separation, VLANs are used. Orchestration of network is possible through the OpenStack network component, Neutron.

**Wireguard** is fast, secure, and reliable considering the limitations. The VPN is established over the Internet and Wireguard uses UDP; UDP does not guarantee delivery and the Internet can be an unstable network. However, to ensure delivery, a tunneling protocol is used. The tunneling protocol also ensures delivery VLAN to the right network. A VLAN in the cloud should also be present in the physical installation. To tunnel network traffic from the cloud, an **Open vSwitch** (https://www.openvswitch.org/ (accessed on 16 February 2023)) is installed in the cloud and another on-premise. Those Open vSwitch installations are then connected over Wireguard with VXLAN protocol. VXLAN packages has a disadvantage that it can not be fragmented, therefore we must ensure that all packages are within the Maximum transmission Unit (MTU). The **MTU** from the Ethernet standard is 1500 bytes without using Jumbo Frames. As our transmission is over the Internet without MTU guaranties, we must ensure that packets are within MTUs. VXLAN has a 50 byte overhead and Wireguard has some overhead, 80 bytes for IPv6 and 60 bytes for IPv4. We must ensure that when network nodes transfer that they do not exceed the MTU boundaries. The network adapters in the solution must obey lower MTU requirements, these settings can often be set in the DHCP server. The use of the protocol GRE in stead of VXLAN might solve some of these fragmentation and MTU issues. Still, the issue of 1500 byte MTU limit on the network out of our control may exist, fragmenting and assembling frames require processing time and power, the optimal solution is to keep the MTU value below the threshold for fragmentation.

Monitoring most of the network traffic in the cyber range is possible through the Open vSwitch as it is a central component forwarding traffic between the physical and virtual environment.

OpenStack Ironic has plugins to manage physical switches as well as virtual switches. Provisioning, running, managing, and cleaning can be made with the support of Ironic.

#### 4.4.5. Emulating IoT Devices

One possibility for emulating IoT devices is through **FirmAE** [39]. FirmAE shows a high degree of successful emulation even without creating and adapting the hardware peripheral emulation. FirmAE is based on Firmadyne [40] and is designed for Linux-embedded devices. Support for other IoT device emulation is possible on QEMU; however, hardware emulation is challenging. Feng et al. [41] suggests a solution that looks promising for emulating purposes, but complete system emulation often requires developing the peripheral components outside the CPU, such as Osman [42] achieved in his thesis. Osman’s solution could be used to transfer input and output signals from the emulated world to the physical, but the delay must be accepted. Some SoC and board implementations exist, but the emulation development cannot cope with the speed that developers create new hardware.

QEMU is the emulator used by most of the literature studied in this project; however, it has shortcomings, as stated earlier. Emulating the Atmega 328 board used in this project completely was not possible since the 8-bit timer for AVR boards was not developed in QEMU. This resulted in the use of timing functions, such as measuring time and delay, would not work. The firmware would run, but tests based on time measurements would fail and the program would not behave as expected. Many other emulations, also in FirmAE, will run, but the behavior can be unpredictable. To have exact behavior in emulation, all peripheral functions need to be developed and it does not seem feasible. Future developments in **AI** will possibly overcome these challenges, as also stated from one of the respondents in the survey, but this is out of scope for this project.

#### 4.4.6. Managing Physical Components

Provisioning the physical IoT devices for an exercise require a different approach. During an exercise a devices can be altered or die. For emulated devices this is probably not a big challenge as a fresh image can be restarted. Many IoT devices and their architectures have implemented some debugging capabilities. Serial UART, JTAG, SWD, and SPI are some capabilities often used in rescuing bricked devices. Serial interfacing gives a large flexibility when the system installed has tools and commands to do tasks. An optimal solution would be to re-flash every devices to make them ready for a new exercise. There are some challenges with flashing devices, NAND flash technology devices wear out with several new writes. Transferring images to flash over UART can be time consuming.

Generally, one has to be careful connecting electrical interfaces to others. Voltage differences creates electrical currents which can damage equipment. In general voltage level shifter should be used, but as the components used in this installation all had 3.3 V interfaces this was not necessary. However, electrical separating the devices reduces the chances of propagating damage if one device has failure. How one device is reset, is depending on the device. Normally all devices have a reset button to go back to factory defaults, but if the factory defaults are compromised it has no value. The reset buttons are often connected to an input on the controller, and is therefore dependent of the system to be running the firmware that handles the button press. Resetting through UART interface requires the device to be ready for receiving and processing commands. At a more low level, if using JTAG, JTAG needs to have the correct capabilities and to be enabled. JTAG can be disabled by the manufacturer in many different ways: it can be disabled in software, only available during a specific time, e.g., during boot or fuses deliberately blown after production test to disable JTAG or SWD functions. SoC with multi-function pins can also combine JTAG pins with other pins and that the selection is made, e.g., during boot.

To handle hardware controlling scripting in Python for controlling the **GPIO** interfaces in Raspberry Pi was chosen. Raspberry Pi is an affordable one-board computer with very flexible use.

To make the Raspberry Pi provisioning automated and fast the Raspberry Pi has to be reprogrammed to boot from network. For this pipxe (https://github.com/ipxe/pipxe (accessed on 16 February 2023)) is used to give Ironic better control when booting the Raspberry Pi. Neutron has to control the network to switch the network to provisioning during boot. Controlling the power cycle of the devices is possible through Ironic power control, but for this project those features were not available. One could use power control through Raspberry Pi GPIO ports and relays and IPMI scripts and interfaces such as diy-ipmi [43]. For this project the use of consumer laptops no managing interfaces on the computer is available. When using relay managed power through, e.g., SNMP (https://docs.openstack.org/ironic/latest/admin/drivers/snmp.html (accessed on 16 February 2023)) supplies the batteries of the laptops need to be removed to ensure that the device is powered off before restoring the power.

Raspberry pi has only one hardware **UART** compatible port through GPIO pins 14 and 15. To handle this limitation for several serial connections it is possible to use other GPIO pins for software serial communication, also called bit banging. As the GPIO ports are not that fast and do not provide hardware buffering, it is a challenge to communicate with other devices that have preset communication speed that exceeds the speed of the Raspberry Pi. The soft_uart (https://github.com/adrianomarto/soft_uart (accessed on 16 February 2023)) project recommends speeds below 4800 bps, which is very low considering that console communication speed through, e.g., Cisco and Linksys routers is at 115,200 bps. Using a real-time operating system (RTOS) could possibly reduce the challenges of timing with bit-banging, but it is not tested in this project. Another solution is to add more USB to serial port (RS-232), but the differences in voltage levels must be handled as discussed before with voltage level shifters. The RS-232 standard was developed 60 years ago and naturally do not follow to days TTL and CMOS standard voltage. Low level on RS-232 is between −5 v and −15 v, while the high level is between +5 v and +15 v. RS-232 has a 2 v fault tolerance, resulting in accepting a low state with voltage lower than −3 v and a high state with voltage higher than +3 v. The circuits used in this project uses 0 v as low and 3.3 v as high; the voltage difference must be handled to ensure the survival of the devices. USB to UART devices are also available cheaply, the voltage level is specified and are provided for 5 v, 3.3 v, and 1.8 v. There even exists USB UART devices with voltage selectors and adaptable voltages and USB devices with several UART channels. The Raspberry Pi can also be extended with more UARTs using the i2c communication channel from the UARTs to Raspberry Pi, these extension boards are a cheap alternative while saving USB ports for some other uses.

#### 4.4.7. Linux Boot Loader

Boot loaders of Linux embedded devices might support booting alternative boot loaders uploaded through UART, network or in attached storage, e.g., Das U boot (https://www.denx.de/wiki/U-Boot/ (accessed on 16 February 2023)) boot loader can load the image through TFTP protocol and write data to memory on the device. Since the boot loader runs before the installed operating system, this gives an opportunity to change the system before it is running and also write desired firmware or configuration before booting. To ensure isolation between administration, provisioning, and exercise areas, the devices must be moved to a different network segment before restarting, this ensures separation of the devices at the physical layer in the **OSI** network model. Resetting devices can be accomplished with power controlling a relay(s) through Raspberry Pi **GPIO** pins. Monitoring and controlling the behavior of the boot loader can be controlled by scripting the serial communication using the devices **UART**. Controlling the serial communication can be accomplished with the use of the **EXPECT** library for python and/or bash. Moving the device to the correct networks can be accomplished by moving physical NIC ports on Open vSwitch bridges via OpenStack Neutron, or in telnet/cli to program managed switches. Further, the managing instances can provide TFTP service to the boot loader.

1. Connect and monitor the serial interface.
2. Power off and on the device with relay connected to Raspberry **GPIO** pin.
3. Stop the boot process of the target through serial interface.
4. Move the target device to the provisioning network by managing the connected switch.
5. Provide TFTP service to the target, configure IP settings and move the TFTP server to provision network.
6. Load image from TFTP server to the device using serial commands.
7. Boot the target device with the new firmware.
8. Move devices to the correct network segments by managing the switches.

#### 4.4.8. Monitoring

Monitoring events and traffic gives valuable information for evaluating an exercise. Information that can be used for creating a timeline of events, timing of events for scores, proof of claims from the participants, etc. Events can also be triggers for dynamic events in a scenario, e.g., an attack is launched when a user logs into a specific server. The Elastic stack (https://www.elastic.co/elastic-stack/ (accessed on 16 February 2023)) is a combination of Elasticsearch, a search engine, Kibana, a user interface for searching and visualization of data, and Logstash, a log collector. Logs can be collected with Beats from Elastic as well. Collecting logs from the management infrastructure is a straight forward task as the management servers have full access to all resources. The network traffic is also possible to duplicate through the central network components. Collecting information from the exercise nodes(red/blue team) is more challenging. The nodes could be configured to push traffic, but this raises some challenges: during the exercise the nodes configuration change resulting in not pushing information, the traffic can be subject to manipulation or blocking and the nodes receiving the information can be subject to attacks. Pulling information can also raise challenges: the pull access is blocked by participants, the pull itself generates log entries in nodes, traffic managing through firewalls are prone to configuration errors and so forth. The monitoring information should preferably be collected out-of-band of the exercise. The cloud solution gives some opportunities to use shared storage and objects which can be used, but this is not a possibility with the physical devices. When the devices log via the **UART**, one can use that capability, however the exercise participant might manipulate the devices so that this function is out of play. The manipulation might not be intentional, but inadvertently. Some policies for the cyber range use could solve this challenge to some extent.

#### 4.4.9. Management

Managing the cyber range should be conducted as a part of the portal, to ease the administrative tasks. The use of OpenStack gives e.g., the possibility to use Horizon as a management tool, logging to a ELK give data presentation via Kibana. As this cyber range project is mainly focusing on the run time environment, this part is not implemented.

Although not implemented in this project, Moodle is suggested as a portal. Moodle is modular and gives possibilities to create modules for uploading and running scripts for executing tasks on the cyber range. Moodle can be used for access control, and thereby separating the teams, and give resource access to instances, such as console, ssh, etc. for the resources allocated. The implementation in this project is made through scripting in Ansible, Terraform, bash, and Python.

#### 4.4.10. Scenario

**Figure 4:**
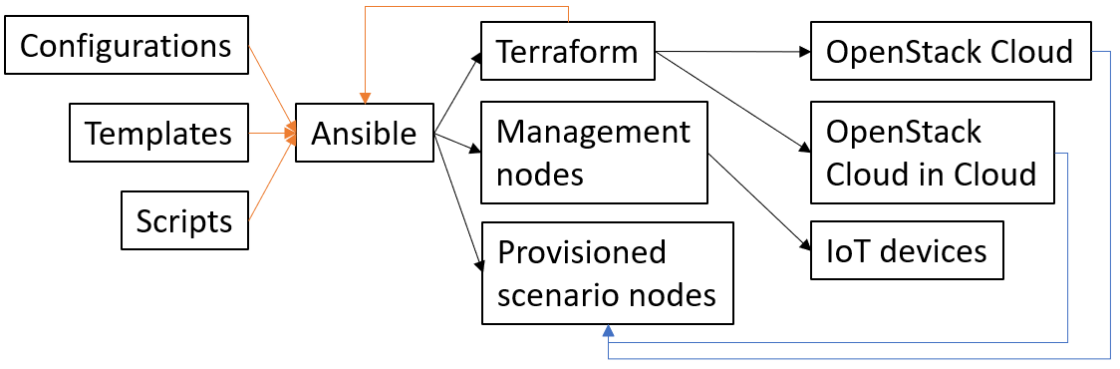
Work flow in creating a scenario.

Scenarios can be developed with the combination of Ansible, Terraform templates, and scripts, utilizing the infrastructure available as shown in Figure 4. The Ansible playbooks, Terraform templates, and scripts are grouped into two collections. One for management (instantiating the cyber range with management resources) and one for the scenario. The scripts must be adapted to the cyber range, e.g., using networks available in the OpenStack infrastructure to provision resources to the correct networks.

Ansible calls on Terraform to provision nodes for the scenario, using defined templates. Ansible polls Terraform for information on how to reach the nodes, waits for the nodes to be ready and runs playbooks to configure the nodes. The management nodes are responsible for resetting and configuring the IoT nodes that cannot be controlled via OpenStack. After Ansible has completed the configuration of each node, the node must be moved to its respective network. When all nodes are installed, configured, and placed in the right network segment, the scripts and playbook complete and the cyber range is ready for an exercise.

In a management host, OpenStack2, FirmAE with a Linux IoT image, is executed inside a docker container. This docker container has network connection to the blue team’s network, thus having access to an emulated IoT device through docker with network address translation (NAT) within the docker container.

If a portal was to be implemented, a descriptive file would be necessary to give present the resources in the scenario, give access to the resources, describe the scenario, and provide assignments to the participants.

### 4.5. Design Discussion

**Figure 5:**
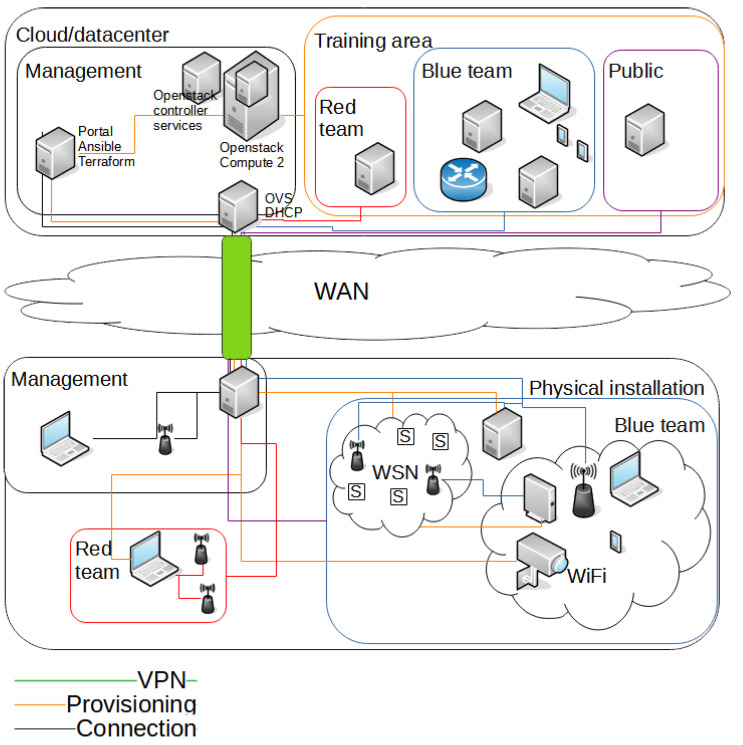
Design of IoT cyber range.

Figure 5 shows the design proposal. Both the physical installation and the virtual installation is divided into at least three areas: red team, blue team, and management. The areas for the red and the blue team can be further divided if the scenario requires more network zones, such as DMZ or further segmentation of networks, etc. To ensure the isolation requirement so that malware or attack traffic is not inadvertently propagated to the Internet, the Internet services on the public zone is emulated by inetsim (https://www.inetsim.org/ (accessed on 16 February 2023)).

OpenStack Neutron provides virtual routers and virtual networks to route traffic between the network segments. As the cloud solution does not guarantee to provide bare metal services, a separate cloud is established within the cloud. The internal cloud is also based on OpenStack and provides services such as identity management (Keystone), image management (Glance), bare metal provisioning (Ironic), networking (Neutron), and virtualization (Nova). OpenStack Ironic provides possibilities to provision hardware through orchestrating network ports through Neutron, images through Glance, powering on/off devices, and network booting. The OpenStack controller contains Neutron controller, Nova controller, Ironic controller, Keystone, Glance, as well as a Nova compute installation to manage the bare metal nodes (compute 1). In the management zone, another OpenStack compute node, compute 2, is provisioned to be controlled by the internal cloud to provision images not available in the "outer" cloud. The compute 2 node purpose is also to emulate any systems with a different architecture than the cloud is built upon, e.g., OpenStack cannot run ARM processor architecture on an x86 hardware, although the hypervisor could have handled such case. Therefore a virtual instance of Linux is deployed so QEMU can perform system emulation of the supported architectures. QEMU then runs an ARM emulation, which installs another OpenStack hypervisor (Compute 3) providing ARM capabilities to the cyber range. Alternatively, the compute 2 node can run FirmAE to emulate Linux-based firmware. The Neutron network between the OpenStack components is handled by a separate VXLAN network via the switch server. The switch server (switch1) has an Open vSwitch installation to manage the networks, team networks, management network, provisioning network, public network, and provides VXLAN capabilities. The same server is also available on Internet to provide VPN access using WireGuard to the physical installation. Depending on the needs for the scenario, nodes to the scenario can be provisioned on the provider cloud or the internal cloud compute node 2 or 3. Compute node 1 is only to provide bare metal services to hardware in the physical part of the cyber range.

On the physical side another Open vSwitch (switch2) installation handles the networks tunneled over VXLAN over VPN. All networks available on the cloud is also available on the physical side. Open vSwitch is able to attach physical network adapters to the desired network, switch2 can also be patched to an external managed switch. The Ironic–Neutron combination on the OpenStack controller can control the network adapters on Open vSwitch and another managed switch, Cisco IOS, is supported through the same plugin.

To ensure monitoring what happens on the physical air, i.e., wireless communication, switch2 also has wireless sniffer(s) installed. As it is not possible to listen to multiple frequencies at the same time, one radio must be installed for each frequency that has to be monitored. A possibility is to scan several frequencies to map what frequencies are in use, but while jumping frequencies information in another frequency might be missed. The red team in the physical installation should also be given possibility to monitor the wireless communication as well as sending signals. Monitoring and sending at the same time is not possible either with only a single radio, multiple radios are required or at least recommended.

### 4.6. Implementation

This section discusses the practical scenario implementation and the hardware used.

#### 4.6.1. Openstack Services

Openstack Neutron provides DHCP capabilities; however, the DHCP service only offers addresses to nodes provisioned via Nova. This might be configurable, but since the cloud is out of our control, we chose to provide own DHCP services for the public network and for the management network. DHCP services for the red and blue team network are to be provided by the team itself or via the scenario definitions. In general, not only for DHCP, it can be a good idea not to be dependent of any special configuration from other service providers.

#### 4.6.2. Physical Installation

The devices used in the implementation are diverse. These are devices used in the project to illustrate all the different challenges and opportunities that can arise. This selection shows some of the diversity in IoT devices, and to some extent, the flexibility of the cyber range. Devices used and tested in this implementation:

- Linksys E900 N300.
- Vivotek FE9180-H.
- Raspberry Pi 3b+ with additional USB-**UART** adapters.
- Aqara SSM-U01.
- Cisco 2960.
- Arduino Pro Mini with Digi XBee S2.
- Laptop with extra USB network adapters.
- CC2531 USB Zigbee traffic sniffer.
- Sonoff Zigbee 3.0 USB dongle.
- Nedis WIFIP130FWT.
- Cleverio Smart switch 51701.

The **Aqara SSM-U01** is a Zigbee switch module. Looking inside the SSM-U01 reveals already marked testpoints for ground (GND), RX, and TX. The switch is powered through mains and handling it requires caution to not touch parts that can have high voltages. Ensuring the RX and TX voltage levels to be the same as the testing device, it requires time to communicate with the device. The SSM-U01 seems to follow the Silicon Labs ZigBee Application Framework CLI language (https://docs.silabs.com/d/zigbee-af-api/6.9/cli (accessed on 16 February 2023)). Some testing gives indication on what commands can be used for resetting, connecting, etc. Useful commands in this device are:

- Plugin network-steering start 0.
  Starts scanning and joining a network. The device has no interface for providing keys, therefore the network must be open for joining and providing the network key. This window of opportunity is where an adversary should listen for traffic.
- Network leave.
  Disconnects from the Zigbee network.
- Reset.
  Restarts the device.
- keys print.
  Prints the network and the link key.

**Figure 6:**
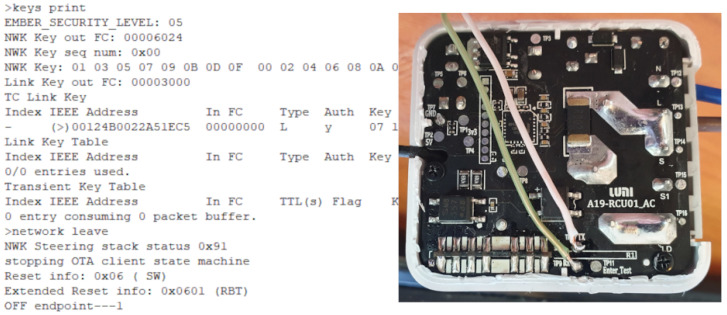
Aqara SSM-U01 switch screenshot and wiring.

Figure 6 shows the connections to the Aqara sensor as well as the output of some commands.

Some Tuya devices with WB2S modules from Nedis and Cleverio were also tested. The WB2S (https://developer.tuya.com/en/docs/iot/wb2s-module-datasheet?id=K9ghecl7kc479 (accessed on 16 February 2023)) is a module with WiFi and Bluetooth integrated and 2 UART devices. According to [44], the first UART (1RX and 1TX) is for programming, and the second UART (2RX and 2TX) is for logging to serial from the SoC. Connecting to the first UART gave no communication on the Nedis WIFIP130P nor the Cleverio 51701. It is possible that the firmware on these devices are programmed not to use the first UART for any communication. There are no other peripheral controllers in the device that require serial communication. The devices have other test points that indicate I/O interfaces, but since the firmware is not available for flashing the device there were no further exploration of the devices.

After soldering a wire on the test point for the second UART unfortunately on the Nedis device, a careless move, resulted in pulling off the 2TX test point on the device, making the device useless for further testing and implementation.

**Figure 7:**
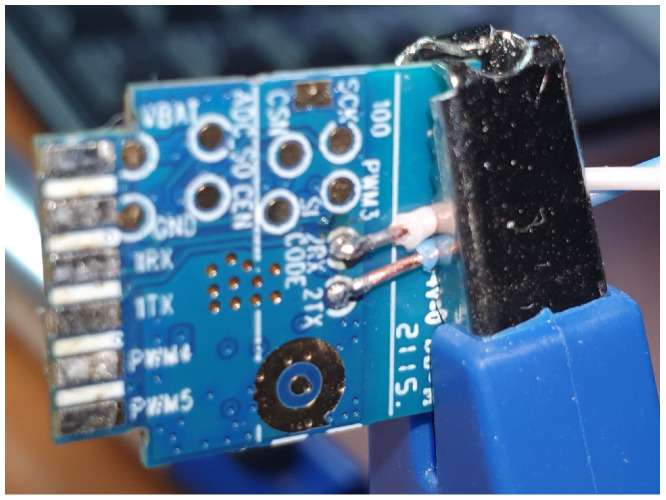
WB2S board dismounted from the Cleverio 51701.

The WB2S board on the Cleverio device is located with its back towards the relay, and mounted inwards into the frame, making it difficult to use the test points. The WB2S module was removed from the device to gain access to the test/soldering points, see Figure 7. The devices PCB also has high voltage connected and special caution is required. Connecting to the 2. UART (2RX/2TX) on the device shows the log of the device, but the device does not seem to accept any commands.

Earlier versions of Tuya devices were possible to reflash with custom firmware over-the-air, but with a new PSK format, that is not possible at the moment (https://github.com/ct-Open-Source/tuya-convert/issues/483 (accessed on 16 February 2023)).

**Vivotek FE9180-H** is a 180° fisheye camera. The camera is powered over the network cable with Power-over-Ethernet (PoE). When opening the camera cover, it reveals test points fairly accessible, many of them are marked. There is a 4-pin connector on the PCB, which seems to be a good candidate for a serial connection. The serial connector often has a VCC, RX, TX, and GND. Measuring the pins with a multimeter shows that is has pin with 0 Ω to testpin marked ground on the PCB and one pin with 0 Ω to testpoint marked 3.3 v on the PCB. The two other pins have at least 1 kΩ resistance to both 3.3 v and to ground. Measuring the voltage shows 3.3 v on all pins except the GND pin. The two other pins, the middle ones, are likely to be TX and RX: one of them fluctuates during boot and is possibly TX as it is likely to do this when writing information from the boot process.

**Arduino Pro Mini** is a programmable micro-controller with at Atmega 328p **SOC**. The SoC supports digital and analog inputs, digital outputs, has **UART** and **SPI** interfaces, and more. For this project, this controller is programmed through SPI. The Arduino is used in combination with **Digi XBee S2** as a ZigBee interface for the Arduino as an IoT device. The XBee has data input and output with serial communication, the same I/O can be used to configure the XBee. Programming the XBee is performed by sending a configuration software to the Arduino, where as the Arduino programs the XBee. When the Arduino has programmed the XBee, the Arduino is programmed with the software for the scenario. To program the Arduino, the SPI interface is used through Raspberry Pi with bit banging using the avrdude ( https://github.com/avrdudes/avrdude (accessed on 16 February 2023)) software. An alternative to program the XBee is to intercept the serial communication line between the Arduino and the XBee while ensuring that the Arduino does not communicate on the same line (e.g., by pulling the Arduino reset pin to low/gnd). The XBee is configured with the Digi XBee supported AT commands. Flashing new firmware and/or changing the operation mode (coordinator/router/end device) is only possible through a XBee Explorer board.

**Figure 8:**
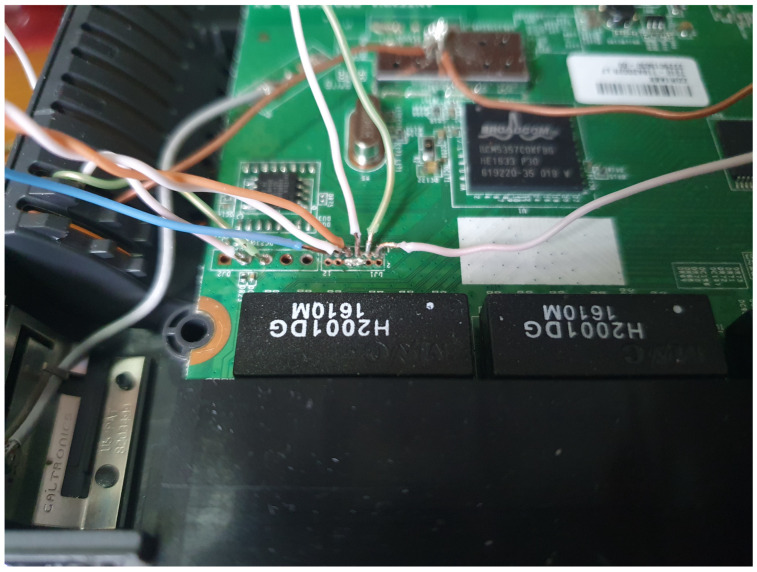
Connection on Linksys E900 N300.

A 12-pin dual-in-line test points (DJ1) and a 4-pin test test point(DJ2) are available on the **Linksys E900 N300**, see Figure 8. The 4-pin is likely to be serial connection and the 12 pin could possibly be JTAG. Measurements show half of the pins to be ground/0 v and others to have some function. JTagEnum [45] is a JTAG scanner for Arduino and Raspberry Pi. Connecting these pins to the Raspberry and doing the scan reports the pin functions. Measurements and results are in Table 1. Pins 2, 4, 6, 8, 10, and 12 are all most likely to be ground.

After identifying the test pins to control the JTAG TAP **Open On Chip Debugger** (OpenOCD) is used. OpenOCD (https://openocd.org/ (accessed on 16 February 2023)) is an open source software for providing debugging, in-system programming and boundary-scan testing for embedded target devices. OpenOCD can debug devices through debug adapters connected to debug port, such as **JTAG** or **SWD**, on target devices. The configuration of OpenOCD has to configure the debug adapter, which in this, case is Raspberry Pi, and configure the target. OpenOCD is capable of resetting devices, read and write to input/outputs, to memory, and to registers. Using this, we can rewrite the IoT device memory with the desired firmware or settings and reboot the device so that the cyber range can restart at a desired state. The Linksys router, and many other Linux-based embedded devices, that use boot loaders with TFTP capabilities, also have the capability to flash the firmware while only starting the boot loader. For devices without flashing capabilities with JTAG or SWD, using the bootloader, UART, and TFTP solves the problem just the same. The Raspberry Pi controls a relay, through GPIO output, the relay is connected to the 12 v power supply of the router.

For connection to the cyber range in the sky, a commodity laptop is used. The laptop must be preinstalled with a Linux operating system, in this case Ubuntu, with WireGuard to connect with the rest of the installation in the virtual realm. To distribute the networks Open VSwitch is used. For full utilization of the Ironic functions for provisioning each device should have their own port in a switch, either on the laptop with Open VSwitch or as in this case a Cisco 2960. Ironic supports both with the networking-generic-switch plugin available for Ironic. As the laptop is not connected to the installation server it has to be installed manually, scripts are provided. VXLAN does not allow fragmenting, other tunneling protocols should possibly also be considered.

**Texas Instruments CC2531 is** a **SOC** with Zigbee capabilities. This is on a USB dongle is used as a sniffer to log Zigbee traffic. The CC2531 must be flashed with new firmware to be a sniffer. The flash _cc2531 (https://www.zigbee2mqtt.io/guide/adapters/flashing/alternative_flashing_methods.html (accessed on 16 February 2023)) software is used for flashing the sniffer via Raspberry Pi. Since the Raspberry Pi only has one UART as default, the implementation also is limited to controlling one device at the time. More **UART**s must be installed when creating scenarios with more IoT devices as discussed earlier.

**Figure 9:**
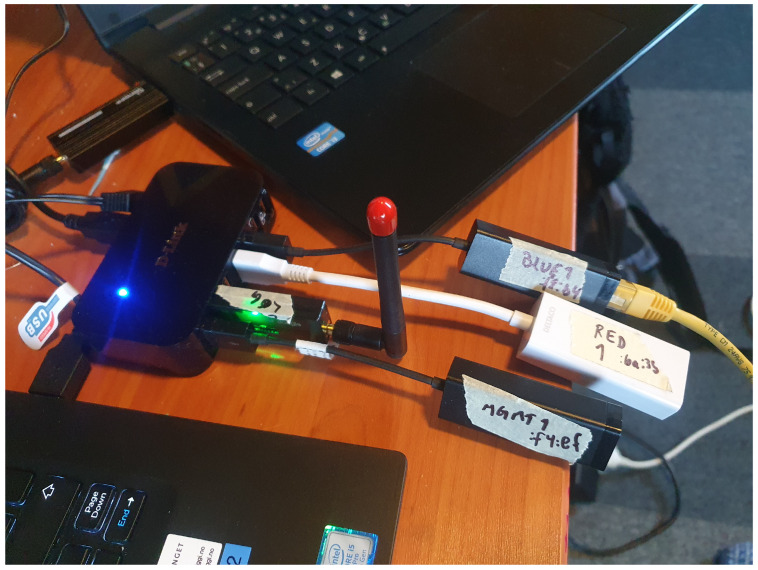
Switch.

Figure 9 shows the switch with three ports, first port on the management network, the second on the red team network, and the third on the blue team network, installed on the laptop in the lower-left corner in the picture. The same computer has the CC2531 installed for logging Zigbee traffic. The other laptop is installed with the Sonoff Zigbee USB dongle as a Zigbee coordinator in the red team segment.

### 4.7. Scenarios

The fourth activity in **DSR** is to demonstrate the artifact. This section describes two attacks towards IoT devices and shows how this hybrid IoT cyber range can implement scenarios with the attacks. The cyber range must be able to implement attacks with relevance to be used in demonstrations, education, etc.

#### 4.7.1. Attack 1: Mirai Botnet

The Mirai botnet was a botnet used to attack among others the DNS provider Dyn. When Dyn was attacked with DDoS the attack resulted in many large sites being unavailable, sites such as Github, Twitter, Netflix, and others. The Mirai botnet is a malware that was designed to target IoT devices. The Mirai botnet uses IoT devices on Internet with available ports and default password to perform an attack. An already infected node scans the network to find devices, and upon finding devices, it tries credentials from a list of factory default passwords. If its malware gains access, then the malware contacts a Command and Control (C&C) server to wait for instructions. While waiting the malware continues to search for new nodes to expand the botnet [5]. The ports scanned by the Mirai bot are port 23 and 2323, and the protocol is telnet. Telnet is a clear text protocol similar to serial communication, but used over network.

The Mirai malware affects Linux hosts and is designed to propagate over networks. The Mirai malware, at least in it original design, was also designed to be run in memory only. When rebooting the device the Mirai code would disappear from the device. If the device still is vulnerable after starting up, it would likely be infected again. The malware have capabilities for DDoD. The volatility of the Mirai infection makes discovering indicators from a Mirai attack difficult on the device itself if the device is rebooted.

The source code for Mirai malware was made publicly available, also on Github. The Mirai botnet has several elements, a C&C server, a database server, scan reciever, DNS server, and a loading server. The domain name for the Mirai botnet is registered in a DNS system and coded into the bot executable. An attacker connects to the C&C server through telnet for control. The C&C server sends commands to infected IoT devices. If an infected devices finds new vulnerable devices, it is reported to the scan receiver. The loading server will act upon the new information and infect the vulnerable device. The new bot then registers itself to the C&C server. The IP address to the C&C server is resolved from the DNS server. The availability of the source code has resulted in many versions of Mirai malware.

For this setup, the C&C, database/MySQLl, scan receiver, and DNS server were installed on Ubuntu servers in the cloud installation. In addition, router and camera firmware were running on the cloud as well as on the physical installation. Ansible, with the help of Terraform and some scripts, was responsible for the orchestration to create, destroy, and recreate the scenario. To make the Mirai network spread to one device, it had to be infected as part of the scenario. The infected device and a vulnerable device were started with a FirmAE installation. As we are interested in the network access and the operating system of the IoT device, FirmAE should be able to provide the functions needed for creating a scenario.

Mirai C&C server is programmed in Go. The bot is written in C and has to be precompiled to each architecture is needs to be executed on, the IoT devices is not likely to contain compilers, nor have the processing power to compile within reasonable time. Compiling on the target on the IoT device would also affect the IoT device performance and thereby increase the chance of being discovered. To cover most architectures for IoT devices the Mirai code was cross-compiled for 10 [46] different architectures. Creating cross-compilers is described at osdev.org.

Files can be inserted to the scenario within images. For instances within the cloud environment cloud-init (https://cloudinit.readthedocs.io/en/latest/topics/examples.html#writing-out-arbitrary-files (accessed on 16 February 2023)) is a possibility. Cloud-init is a method for cloud instance initialization, also supported by Openstack. For devices outside the Openstack environment configuration is possible through Ansible. Some systems may have user data limit, such as AWS has a 16 KB size limit, making Ansible the more prominent solution even for cloud instances.

The purpose of this test is to confirm botnet propagation from the cloud infrastructure an to the physical side. In addition, to revert the changes the botnet may do.

FirmAE establishes a serial device as a Unix socket. This Unix socket can be used as a serial port to the emulated device and act as a side channel for configuring the IoT device. On the host FirmAE creates network devices via QEMU, the devices are available as tap Ethernet devices on the host and in the same network segment as the emulated device. The Ethernet device can be added as a port to the Open vSwitch installation with a tunnel to the cyber range network. Using the correct vlan id when adding the port will make the emulated device be available on the correct network in both the virtual and the physical part of the infrastructure.

#### 4.7.2. Attack 2: Zigbee

**Figure 10:**
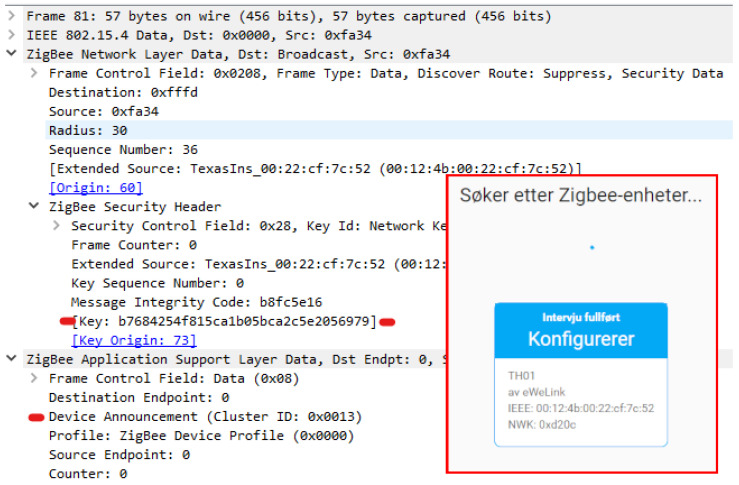
Screenshot Wireshark–Zigbee network key while configuring the device in Home Assistant.

As described earlier, Zigbee is vulnerable for eavesdropping of the network key while linking new devices to the network. An installation with the Sonoff Zigbee 3.0 dongle integrated into Home Assistant will make Home Assistant be able to link to new Zigbee nodes. The default setting of the Sonoff Zigbee dongle is to allow linking for all devices, meaning that the network key is also available for all nodes within reach. Trying to link a new device to the network will transmit the network key over the air and be available for sniffing with whsniff (https://github.com/homewsn/whsniff (accessed on 16 February 2023)) using the CC2531 USB device and viewing the packets in Wireshark/tshark. The default global trust center link key must be inserted into Wireshark to decrypt the traffic when the network key is transferred, and the network key is revealed as shown in Figure 10.

With the network key available the traffic content is available for listening, interception, and inserting. Controlling a relay for light might not have a big impact, but for devices that control devices with higher energy potential, such as heating elements etc., the devices could cause fire if overheating, water heaters might explode if the water reaches boiling temperatures and so forth, where the result might be fatal.

The un-secure approach for network key exchange is a result of that the installation must be as easy as possible for the end user while pairing the devices, and since the devices often do not have an interface other than one single button. The simplicity increases the usability and reduces cost, but comes with a cost of reduced security.

## 5. Evaluation and Discussion

The last activity in **DSR** is evaluation. The main goal of the evaluation activity is to assess whether, and how well, the designed artifact solves the problem. However, the product is also compared to the requirements as defined in Section 4.2.1, on page 11; these are used as guidelines when developing the cyber range in this work. Some requirements are evaluated *ex ante*, by informed argument, as they are not part of the development, as opposed to the requirements that are developed, is evaluated *ex post*, by doing an experiment.

The main problem to be solved in this work is to reduce the resource usage for doing IoT cyber exercises, mainly resources in form of labor hours. Evaluating this project in large scale is difficult as there are not that many IoT devices available at hand for deploying. One can safely assume that several processes can be executed in parallel depending on the managing channel count available. Time measuring evaluation is performed by testing the artifact, running the scripts developed in the project, and comparing it to the time it would take to manually set up the same exercise environment.

In [15], a cyber range with 75 virtual machines and 5 networks were deployed in 5 min after the scenario files were prepared and uploaded. The assumption for measuring time is that all files and connections are prepared. Deployment requires timing and orchestration as some components can not be deployed or prepared before some other components. Physical components in this hybrid environment has other limitations that virtual systems do not have.

This work has used of-the-shelf IoT devices as well as self-built devices. The IoT world has a plethora of brands, devices, and solutions. The validity in the tests are considered to be good as the cyber range has support for both handling emulation as well as many different types of physical components. The setup can therefore be complex, the work discusses some of those challenges, but to handle as many types as possible the setup has to be complex.

While some papers in the related work section present testbeds and cyber ranges, also for IoT, none of the papers have a complete description on how it could be implemented, testing, and finding possible technologies, nor have an approach for using IoT test access points for preparing a cyber range and monitoring the components.

### 5.1. Requirements

The requirements and their fulfillment for this IoT hybrid cyber range are listed in Table 2. A requirement can be fulfilled in design or in implementation. When covering a requirement in implementation, it is implied that the requirement is also fulfilled in design. To recap the requirements:

- Flexibility, scalability, adaptability, interoperability.
- Shareability and open source.
- Fidelity.
- Isolation, safety, resilience, and reliability.
- Cost-effectiveness.
- Built-in monitoring.
- Easy access, usability, and user interfacing.
- Service-based access.
- Heterogeneity, handle diversity in IoT, and emulating digital twin.
- End-to-end testbed.

#### 5.1.1. Flexibility, Scalability, Adaptability, and Interoperability

Combining the flexibility in OpenStack services with the physical components, and linking these with Open vSwitch, WireGuard, and VXLAN functionality gives a large flexibility in how to design scenario networks, how the networks are linked, and how they can be combined.

#### 5.1.2. Shareability and Open Source

To cover the requirement for shareability, the project has focused on using open source software. Implementations on this cyber range will be published on GitHub. The software used in the implementation is all open source.

#### 5.1.3. Fidelity

The suggested taxonomy and unified functional component architecture from Yamin et al. [2] is used as a reference for this cyber range. Well-known large software projects are used as a base for the infrastructure. By this, the fidelity requirement is covered.

#### 5.1.4. Cost-Effectiveness

The cost for implementing this cyber range is reasonable. The devices used are low-budget devices. Cost-effectiveness is not only about investing devices, reducing work hours for administering the cyber range will also reduce costs. Using provisioning, scripting, and interfacing with the IoT devices will automate tasks and thereby reduce time spent resetting already developed scenarios.

#### 5.1.5. Isolation, Safety, Resilience, and Reliability

Using side-channels, such as JTAG and serial interfaces, to communicate with the IoT devices ensures isolating the management segment from the exercise/training/testing segment of the cyber range. Simulating our own internet services, providing services on our own cloud, and isolating the exercise segment our services from other networks and the Internet. Any attack happening in the training area should not affect the managing segments nor be spread outside the exercise area provided the provisioning steps are carefully considered. It has also been suggested to use specially built rooms or areas to ensure that wireless signals from outside are not present in the cyber range and that the wireless signals whit in cyber range are not propagated to the outside world.

#### 5.1.6. Monitoring

This design suggests methods and solutions for monitoring, but it is not implemented nor tested in this work. Monitoring through the side-channels in IoT gives an opportunity to log what happens in every device, and also use the same timestamps as the rest of the cyber range. One alternative to extract the information live, directly from an IoT device is to collect the logs after the exercise, which can result in only partial logs due to storage limitations, or compromised logs due to an action by the training teams in the exercise.

#### 5.1.7. Easy Access, Usability, and User Interfacing

A portal was outside the scope of this work, the design suggests the requirements for user interfacing, usability, and easy access to be a part of the portal. Finding and accessing the side-channel interfaces on the IoT devices can also be a challenge due to the design and lack of design documents of the devices, but the approach and used tools for this tasks are referred to in the report.

Selecting IoT devices for use in a cyber range for exercises must be performed with care. To be able to automate a cyber range provisioning the IoT devices must have side-channel interfaces, which can be used in automation and monitoring. JTAG/UART/SWD/GPIO/SPI is used in this project. If the cyber range is used as a firmware testbed, emulating with FirmAE can be sufficient. Extracting from or writing to memory can be made with test access ports. Writing new firmware to the physical router used in this project was possible since the router starts a TFTP server for a brief period during boot. This was a bootloader feature in this firmware. Features in firmware depends on the developer.

#### 5.1.8. Service-Based Access

Service-based access is out of scope for this project, all though most of the components used provide service-based access. The overall design does not limit it, but no component was given the role for this.

#### 5.1.9. Heterogeneity, Handle Diversity in IoT, and Emulating Digital Twin

FirmAE adds the cyber range a capability to emulate a large library of Linux-based IoT firmware’s. Because of the challenges in emulating all peripherals and integration’s of an IoT device, this cyber range also has a physical infrastructure, which is capable of handling real IoT devices provided they have interfaces for managing the devices.

#### 5.1.10. End-to-End Testbed

The cyber range do not have any limitations that prevents creating scenarios or environments that can simulate end-to-end connectivity, from IoT device to cloud services. The infrastructure has support for implementation from local network, where the home IoT devices operate, to a simulated Internet or public network, where the cloud services reside.

### 5.2. Efficiency

Even with the knowledge on how the devices work, a manual setup with 10 devices, installed for the networks, logging in, and installing configuration, and software, it is difficult to complete all tasks for this implementation in under one hour, even for this small setup. The total time would of course be reduced if the preparation is made by several persons doing parallel tasks, however, some time must be set to coordination. Using the scripts and design developed in this project, the same tasks are performed in less than 40 min. Time measurement is not used as an exact measurement, since factors such as network speed, processing power, wireless scanning algorithms, and so forth can affect time usage. To ensure reliability in the efficiency, the cyber range is reset to the same scenario several times and reporting the average.

The single most time-consuming task is to provision the bare metal computer via OpenStack Ironic. Transferring the image to the boot image on the computer takes time. The time consumption is depending of the network speed, disk speed, and processing speed in both ends. Even if the time difference in doing it manually vs. automation is not that large, the automation ensures that the setup is the same for all reruns if the same scenario is to be executed again. From the deployment start, until Ironic reports the node active, it takes about 30 min on a laptop computer with Intel Core i3 3217u with 4 GB RAM and 500 GB SATA 5400 rpm HDD, to install a Ubuntu Bionic with an image size of 642 MB. It is first at this point that the client is ready to have additional software installed, unless software is already prepared in the image. Doing software installation as a separate part, after the image installation, gives more flexibility. As WireGuard and VXLAN both use UDP, and as UDP is an unreliable protocol, network congestion in some part of the transmission chain could affect performance.

## 6. Ethical Considerations and Limitations

The software used in this work is used within the license requirements to the best of our knowledge. Anyone using the same software is responsible for accepting the license of the software. Care must be taken when implementing a cyber range to ensure that the range is isolated from other systems to ensure that no harm is propagated outside the range. Using a cyber range to learn vulnerability exploitation gives an ethical and moral responsibility to report zero-day vulnerabilities to the manufacturer or maintainer and also not use the vulnerabilities to compromise systems outside the cyber range. While the software for malware is available on the Internet, and there exists a description of how to use them, anyone working with malware must handle them in a controlled environment. A misconfigured cyber range can potentially gain Internet access and spread malware, even if it is not intentional.

The participation in the questionnaire is limited, with only 18% of the 22 initial recipients received. The total number of recipients is also limited. There was a request to forward the survey to others as well, and it was indeed forwarded by some. There was also one respondent who replied about having a busy schedule. The community in cyber range development is small, and people focusing on IoT cyber ranges are even fewer. While the count may not give a full empirical picture of the world, the results do give some valuable information that this work has chosen an approach that others find useful as well, both in design and choice of tools. At least, it also confirms that the choices in this design are relevant. Since the size of the community is limited, it is likely that they have busy schedules and are not available to participate in surveys at every level. As Yamin et al. [15] also pointed out, these are persons with a very specific skill set, and the selection is considered to be small.

During the implementation, there were some challenges with the electronics of the devices. On the UART of the Vivotek FE9180-H camera, the RX pin stopped working. The TX pin still sent data from the camera, but the terminal was not able to send commands to the camera. On the Nedis WIFIP130FWT, a soldering point was loosened from the PCB, making the TX pin unavailable on the device. A soldered wire loosened from the ground soldering point on the Aqara SSM-U01 when moving it around, resulting in a short circuit, and the entire device did not start anymore. These examples show the requirement for caution when working with electronics and how sensitive the components are. When soldering on the test wires, it is recommended to fasten the wires to the device with, e.g., a glue gun. This can ensure insulation as well. Isolating the devices electrically from each other with, e.g., optocouplers should be considered in a production implementation. Limiting potential large currents with resistors is also a good safety measure to have components survive unfortunate incidents.

## 7. Conclusions and Future Work

The hybrid IoT cyber range designed in this work fulfills the functional architecture components and requirements discussed in Section 4.4. The implementation was limited to the scenario and run-time environment components. The requirements surveyed for the cyber range, which are within the scope of this work, have been discussed and found to be covered by the design. When selecting physical IoT devices for use in a cyber range, care must be taken. While a device can be instructed to perform tasks via, for example, the serial/UART side channel, it may not be enough if the device is unmanageable due to corrupt firmware or changes to parts of the firmware. Using alternative communications with the devices offers an opportunity to automate their behavior and log activity while still being able to use the same devices for training, testing, and exercises. However, creating the entire scenario, finding IoT alternative communication capabilities, and creating scripts can be time-consuming. If the exercise is a one-time event, it may be a better option to manually set up the physical IoT part of the environment. This must be taken into consideration before using the approach in this work.

Automating scenario creation not only saves time, but also ensures that the same exercise has the same setup and configuration for every run. This work focused on the integration between a virtual and physical cyber range and how to administer IoT devices out-of-band of the exercise. The other components of a cyber range are not implemented, as they are more general for cyber ranges. The next step is to implement a complete cyber range with all components. The cyber range should also include an approach for emulating non-Linux-based IoT devices, possibly using p^2^im [41]. A more generalized approach for creating device libraries would make it easier to add more devices to the cyber range. However, the diversity in IoT devices makes a general approach difficult. Common elements such as power control, serial communication over UART, JTAG, or SWD usage could be used as elements for function prototyping/interfaces.

## Figures and Tables

**Table 1:** Measurements for 12-pin connector DJ1 on Linksys E900.

Pin	R VCC	R GND	V	Pin	R VCC	R GND	V
1—nTRST	630 Ω	640 Ω	3.3 v	2	100 Ω	0 Ω	0 v
3—TDI	630 Ω	570 Ω	3.3 v	4	100 Ω	0 Ω	0 v
5—TDO	630 Ω	640 Ω	3.3 v	6	100 Ω	0 Ω	0 v
7—TMS	630 Ω	640 Ω	3.3 v	8	100 Ω	0 Ω	0 v
9—TCK	630 Ω	640 Ω	3.3 v	10	100 Ω	0 Ω	0 v
11—nSRST	∞ Ω	∞ Ω	3.3 v	12	100 Ω	0 Ω	0 v

**Table 2:** Requirements evaluation.

Requirement	Fulfillment
Flexibility, scalability, adaptability, interoperability	Design: yes Implementation: yes
Shareability, open source	Design: yes Implementation: yes
Fidelity	Design: yes Implementation: partially
Isolation, safety, resilience, reliability	Design: yes Implementation: partially
Cost-effectiveness	Design: yes Implementation: yes
Built-in monitoring	Design: yes Implementation: partially
Easy access, usability, user interfacing	Design: partially Implementation: partially
Service-based access	Design: partially Implementation: no
Heterogeneity, handle diversity in IoT, emulating digital twin	Design: yes Implementation: yes
End-to-end testbed	Design: yes Implementation: yes

## Data Availability

Data sharing not applicable.

## Abbreviations

The following abbreviations are used in this manuscript:

PhD	philosophiae doctor
CoPCSE@NTNU	Community of Practice in Computer Science Education at NTNU
GCD	Greatest Common Divisor
PCB	Printed circuit board
IoT	Internet of Things
IIoT	Industrial Internet of Things
UART	Universal Asynchronous Receiver–Transmitter
JTAG	Joint Test Action Group
TAP	Test Access Port
SWD	Serial Wire Debug
CPS	Cyber–Physical System
RTOS	Real-Time Operating System
NIST	National Institute of Standards and Technology
DDoS	Distributed Denial of Service
DoS	Denial of Service
IRT	Incidence Response Team
AI	Artificial Intelligence
MTU	Maximum Transmission Unit
GPIO	General Purpose Input/Output
OSI	Open Systems Interconnection
SoC	System On Chip
SPI	Serial Peripheral Interface
VXLAN	Virtual Extensible LAN
DSR	Design Science Research
ADB	Android Debugging Bridge
